# Time Post-Stroke and Upper Extremity Stroke Motor Recovery Rehabilitation: A Meta-Analysis

**DOI:** 10.1177/15459683251356975

**Published:** 2025-07-23

**Authors:** Sarvenaz Mehrabi, Cecilia Flores-Sandoval, Jamie L. Fleet, Sean P. Dukelow, Emma A. Bateman, Robert Teasell

**Affiliations:** 1 Lawson Research Institute at St. Joseph’s Health Care London, Ontario, Canada; 2 Department of Physical Medicine and Rehabilitation, Schulich School of Medicine and Dentistry, Western University, London, Ontario, Canada; 3 Department of Clinical Neuroscience, Hotchkiss Brain Institute, University of Calgary, Calgary, Alberta, Canada

**Keywords:** Fugl-Meyer assessment, stroke, rehabilitation, upper extremity, motor recovery, time post stroke, meta-analysis

## Abstract

**Background:**

Understanding the impact of timing of post-stroke motor recovery research trials is critical for clinical care.

**Objective:**

To examine and compare differences in Fugl-Meyer Assessment Upper Extremity (FMA-UE) scores at 2 different time points post-stroke on the effectiveness of upper extremity (UE) rehabilitation interventions compared to conventional care or sham therapy controls in stroke rehabilitation randomized controlled trials (RCTs).

**Methods:**

A meta-analysis was conducted in accordance with PRISMA guidelines. Searches were conducted in CINAHL, Embase, PubMed, Scopus, and Web of Science, up to April 1st, 2021. Inclusion criteria were: (1) English RCTs of adults (≥18 years) diagnosed with stroke; (2) examined a single intervention for stroke UE rehabilitation; (3) used conventional care/sham as the control arm; and (4) assessed FMA-UE as one of the outcome measures.

**Results:**

157 RCTs were included, including 17 types of interventions. In the acute and subacute phases post stroke, 16 interventions were assessed, and the analyses of 11 interventions showed significant beneficial effects. In the chronic post-stroke phase, 9 intervention types were assessed, and 7 of them showed significant improvements. Greater FMA-UE score improvements were found for the same interventions in the acute and subacute post-stroke phases when compared to the chronic phase.

**Conclusions:**

Interventions studied in the acute and subacute phases showed greater magnitude improvements in the FMA-UE scores compared to the chronic phase. The effectiveness of upper extremity rehabilitation interventions may be underestimated when studied exclusively in the chronic phase, with some of the observed differences potentially attributable to variations in baseline severity.

## Introduction

Motor impairment is among the most common and disabling consequences of stroke.^1^ Stroke-related upper extremity (UE) motor impairment interferes with activities of daily living, avocational, and vocational activities, leading to increased dependence and reduced social and community participation.^2^ Effective UE stroke rehabilitation that maximizes recovery, functional independence, and quality of life is therefore a priority for patients, caregivers, healthcare providers, and society.^3^

Numerous factors influence recovery from stroke-related UE impairment, including stroke severity, age, and importantly, time post-stroke onset, all of which influence the capacity for neuroplastic change.^4^ After a stroke, motor recovery is most rapid in the acute and early subacute phase, with continued gains typically occurring within the first 6 months, and gradually slowing to a plateau after 6 months.^5,6^

As a result, the timing of rehabilitation initiation plays a critical role in the effectiveness of interventions, and the acute and subacute phases post-stroke represent a critical window for motor recovery in stroke rehabilitation.^7,8^ While clinical rehabilitation almost always takes place in the acute or subacute phase,^7,8^ research trials are most commonly conducted during the chronic phase due to greater feasibility. Understanding how time post-stroke affects responsiveness to rehabilitation is crucial to clinical generalizability and to better interpretation of research findings.

Randomized controlled trials (RCTs) are the gold standard for determining which UE stroke rehabilitation interventions are effective. The main goal of many UE stroke rehabilitation RCTs is to evaluate if an intervention can improve UE motor recovery, and if so, by how much. Given the importance of post-stroke UE motor rehabilitation, consensus regarding the use of outcome measures (OMs) and timing of measurement is critical to compare interventions between trials.^9^ The Fugl-Meyer Assessment^10^ is a core outcome measure recommended by rehabilitation expert panels for evaluating post-stroke motor impairment, given its strong psychometric properties, including high interrater reliability, item consistency, validity, and responsiveness to change.^11-15^ A recent systematic review of post-stroke UE rehabilitation trials showed that out of 1276 RCTs, 48.5% used the Fugl-Meyer Assessment as an outcome measure.^16^ The Fugl-Meyer Assessment Upper Extremity (FMA-UE) is a widely used, objective measure of motor impairment in the shoulder-arm, wrist, hand, as well as coordination and speed in rehabilitation research and clinical practice.^17^

In this meta-analysis, we drew upon the widespread use of the FMA-UE in stroke rehabilitation trials to examine how time post-stroke influences intervention-related changes in upper extremity motor recovery, as measured by the FMA-UE, when compared to conventional or sham interventions in RCTs.

## Methods

Data presented in this manuscript come from a unique database of post-stroke UE motor rehabilitation RCTs. Other parts of this database, different from the content of this manuscript, have been published elsewhere.^16,18-21^

### Main Database Search Strategy

To develop an extensive database of post-stroke UE motor rehabilitation RCTs from inception, systematic searches were conducted in 5 databases: CINAHL, Embase, PubMed, Scopus, and Web of Science, up to April 1st, 2021, in accordance with the Preferred Reporting Items for Systematic Reviews and Meta-Analyses guidelines for systematic reviews and meta-analyses (PRISMA). Additional studies were identified through hand-searching reference lists of “Chapter 10: Upper Extremity Interventions” of the Evidence-Based Review of Stroke Rehabilitation (EBRSR), available at http://www.ebrsr.com/. The detailed search strategy has been published elsewhere.^19^

### Main Database Eligibility Criteria

Studies in English were included in the main UE motor rehabilitation intervention database if they: (1) were RCTs or RCT crossovers; (2) studied adults aged ≥18 years diagnosed and affected by ischemic and/or hemorrhagic stroke; and (3) examined interventions for stroke UE motor rehabilitation as the primary objective of the study. Studies were excluded if they were a secondary analysis or long-term follow-up of an original RCT that did not present new data.

### Database Study Selection and Data Extraction

Search results were imported into Covidence software (Veritas Health Innovation, Melbourne, Australia; Available at www.covidence.org), where duplicates were automatically removed. Two independent reviewers performed title and abstract screening, as well as full-text screening and data extraction, with an independent senior researcher available for conflict resolution. Data were extracted using a custom Covidence extraction template and subsequently exported to online Excel for management.

### Meta-Analysis Eligibility Criteria

For the purposes of the meta-analysis, RCTs in the main UE motor rehabilitation database were reviewed and included if they (1) used the FMA-UE subscale to assess UE motor recovery and reported a total score out of 66; (2) examined a single intervention for stroke UE rehabilitation as indicated in the primary objective of the study in at least 1 experimental arm; and (3) used conventional care or sham therapies in at least 1 control arm. Studies were excluded if: (1) the experimental arm(s) received a combination of 2 or more interventions; (2) the FMA-UE scores were not reported out of 66 (e.g., partial sub-scores of the FMA-UE or the upper and lower extremities scores presented together); and (3) the FMA-UE scores were not reported in an extractable format (e.g., data presented in figures or p values only) or convertible to mean and standard deviation (SD) values.

### Meta-Analysis Study Selection and Data Extraction

For the purpose of the present analysis, data extraction included authors, year, title, number of patients in each study arm, intervention description and duration, control description and duration, time post-stroke, and FMA-UE scores. Study screenings and data extractions were performed by 2 independent reviewers. Time post-stroke was determined as the time from stroke onset until the initiation of the intervention and was categorized into acute/subacute (up to 6 months) and chronic (>6 months). Acute and subacute time points were considered together given the extensive crossover of acute studies into the subacute phase.

The FMA-UE^10^ measures impairment in the shoulder, arm, wrist, hand, as well as coordination and speed of coordination activities. The subscale is administered in ascending numerical order, with 33 items scored 0 to 2 (0 = cannot perform, 1 = performs partially, 2 = performs fully) for a total of 66 points.^22^ Point measures and variability estimates for FMA-UE were collected at baseline, and either post-intervention and/or the change from baseline scores. If available, change scores from baseline were selected over pre- and post-intervention scores. For those studies that separately reported the shoulder, hand, wrist, and coordination subscales of FMA-UE,^10^ the total mean and SD were calculated and included in the meta-analysis. Point measures and variability estimates were converted into means and SDs, where possible, using formulas by Wan et al.^23^

All types of interventions used for post-stroke UE motor recovery were eligible for this study. For multi-arm RCTs that compared different intervention groups with a conventional control group, each arm was analyzed according to the respective intervention. We considered RCTs that had at least 1 arm for UE motor recovery assessing a single intervention compared to a control arm of either conventional or sham therapy. Conventional therapy, a heterogeneous term that is often poorly described in published RCTs,^24^ was defined as medical care and/or rehabilitation that was provided to participants as part of the standard of care in the study setting according to stroke practice guidelines, including words and/or phrases such as: Conventional/ Usual/Standard/Traditional/General/Regular/Routine/Normal/Classical/Physician-prescribed Care/Therapy. Sham therapies included either sham stimulations or sham rehabilitation therapies.

After data extraction and intervention classification, interventions were specifically included for the purposes of meta-analysis if at least 2 different RCTs conducted in the same post-stroke phase met the inclusion criteria, providing at least 2 unique comparisons where an intervention was compared to a conventional or sham therapy.

### Risk of Bias Assessment

The Cochrane Collaboration’s tool was used to assess the risk of bias (RoB) of the included RCTs.^25^ The tool evaluates 6 domains of bias and classifies the risk as low, unclear, or high. Two independent reviewers performed the assessment, and the conflicts, if any, were discussed with a third senior reviewer.

### Statistical Analysis

A meta-analysis was conducted using the “metacont” function from the “meta” package in R.^26,27^ The analysis was based on the mean differences (MD) between intervention and control groups, with the corresponding standard deviations and sample sizes. The MD was chosen as the summary measure to quantify the effect size across studies, alongside 95% confidence interval (CI) (lower CI-Upper CI) and *p*-value. Cochran’s Q test and I^2^ statistics were used to test and quantify heterogeneity across studies. Given the heterogeneity across studies of different interventions, a random effects model was applied. Mean change scores for both intervention and control groups were calculated as the difference between post-intervention and pre-intervention means.

## Results

### Characteristics of the Selected Studies

**Figure 1.**
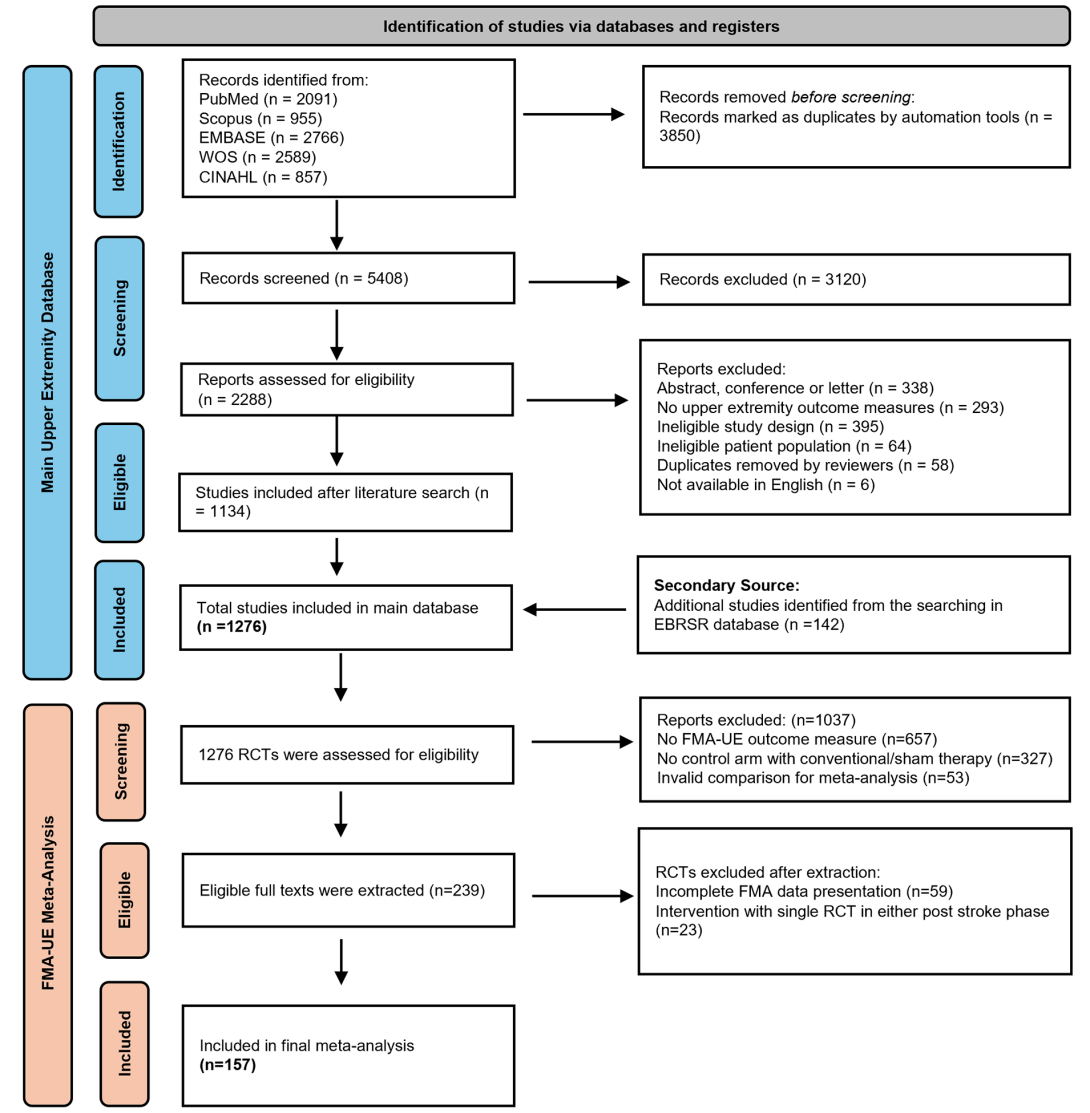
PRISMA flowchart.

A total of 157 RCTs were included in the meta-analysis (Figure 1). Five multi-arm RCTs compared different intervention groups with a conventional control group. Ninety-one RCTs with 96 comparison arms, including 3792 participants, were included in the meta-analyses in the acute/subacute phase, and 67 RCTs with 68 comparison arms, including 2753 participants, were included in the meta-analyses in the chronic phase. One RCT of mirror therapy included subgroups of participants in both the acute/subacute and chronic post-stroke phases, and each subgroup was analyzed within the corresponding phase.

A total of 17 unique interventions were studied across all time phases post-stroke. Of these, 8 interventions were studied in both post-stroke phases, 8 interventions were studied only in the acute/subacute phase, and 1 intervention was only studied in the chronic phase. Table S1 provides a brief description of the included interventions and lists the number of eligible RCTs in which the intervention was compared to conventional/sham therapy. More details of the RCTs included in the dataset are provided in Supplemental Material 1. Table 1 shows the interventions studied and the calculated MD and 95% CI for each treatment versus conventional therapy.

**Table 1.** The Calculated MD and 95% CI for Each Treatment Intervention Versus Conventional Therapy in Acute/Subacute and Chronic Time Post Stroke Phases.

Intervention	Acute/subacute phase		Chronic phase	
	Baseline FMA	MD [95% CI]	Baseline FMA	MD [95% CI]
Action observation	26.9	**5.08 [1.95, 8.22]**	—	—
Acupuncture	23.6	4.02 [−0.17, 8.22]	—	—
Bilateral arm training	31	2.21 [−0.98, 5.40]	45.8	**7.23 [3.15, 11.31]**
CIMT	37.3	**9.02 [2.57, 15.47]**	44.9	**2.72 [1.10, 4.34]**
Mirror therapy	21.9	**4.13 [1.75, 6.51]**	29.6	2.09 [−0.24, 4.41]
Motor imagery	26.1	**6.61 [0.08, 12.43]**	32.3	**4.20 [1.11, 7.28]**
NMES	14.9	**3.85 [0.14, 7.56]**	—	—
RAS	26.4	**6.16 [2.97, 9.35]**	—	—
Robot-assisted training	33.8	**2.35 [0.23, 4.46]**	27.0	**1.55 [1.06, 2.03]**
rTMS-HF	28.6	**9.73 [3.08, 16.34]**	—	—
rTMS-LF	36.9	**5.12 [2.5, 7.73]**	31.2	−0.83 [−4.41, 2.76]
Sensory stimulation	—	—	12.4	**1.65 [1.32, 1.98]**
TBS	25.5	**9.33 [7.05, 11.62]**	—	—
tDCS-C	17.2	5.93 [−5.63, 17.5]	31.4	**4.87 [0.07, 9.66]**
TENS	14.3	3.41 [−0.11, 6.94]	—	—
Task-specific training	13.6	6.59 [−0.9, 14.08]	—	—
Virtual reality	33.1	**2.47 [0.64, 4.32]**	39.3	**2.61 [1.17, 4.06]**

*Note*. Numbers in bold show that the intervention was significantly effective compared to the control condition.

### Risk of Bias

98 RCTs had a high RoB, followed by 54 RCTs with an unclear and 5 RCTs with a low risk. The most common domains with an unclear RoB were respectively “selective reporting” (domain 6, unclear in 67.5% of RCTs), allocation concealment (domain 2, unclear in 61.2% of RCTs), and blinding of participants and therapists (domain 3, unclear in 35% of RCTs). The most common domains with a high RoB were respectively “blinding of therapists and participants” (domain 3, 54.1% of RCTs) and “selective reporting” (domain 6, 12.1% of RCTs). Across all 98 RCTs with the overall high RoB, 61 RCTs had only domain 3 with a high RoB, with other domains as low or unclear. For more details, see Supplemental Material 2.

### Meta-Analyses

Of the 16 unique interventions assessed in the acute/subacute phase, 11 demonstrated statistically significant beneficial effects over conventional therapy based on the FMA-UE score. Three interventions showed the largest treatment effects, with MDs exceeding 9 FMA-UE points: high-frequency repetitive transcranial magnetic stimulation (rTMS-HF; MD = 9.73), theta-burst stimulation (TBS; MD = 9.33), and modified constraint-induced movement therapy (mCIMT; MD = 9.02). Statistically significant effects were also observed for motor imagery (MD = 6.61), rhythmic auditory stimulation (MD = 6.16), low-frequency repetitive transcranial magnetic stimulation (rTMS-LF; MD = 5.12), action observation (MD = 5.08), mirror therapy (MD = 4.13), neuromuscular electrical stimulation (NMES; MD = 3.85), virtual reality (MD = 2.47), and robot-assisted training (MD = 2.35).

Of the 9 unique interventions in the chronic phase, 7 interventions showed a significant intervention effect, including bilateral arm training (BAT; MD = 7.23), cathodal transcranial direct current stimulation (tDCS-C; MD = 4.87), motor imagery (MD = 4.20), virtual reality (MD = 2.61), CIMT (MD = 2.72), sensory stimulation (MD = 1.65), and robot-assisted training (MD = 1.55).

Eight interventions were assessed in both the acute/subacute and chronic phases. Three interventions, CIMT, motor imagery, and robot-assisted training, showed statistically significant beneficial effects in both phases, but a greater MD in FMA-UE was found in the acute/subacute phase. Virtual reality also showed a statistically significant beneficial effect in both post-stroke phases, with a slightly greater MD in the chronic phase. Mirror therapy and rTMS-LF showed statistically significant beneficial effects only in the acute/subacute phase, while statistically significant beneficial effects of BAT and tDCS-C were found only in the chronic phase, even though, with tDCS-C, the actual increase in FMA-UE MD was greater in the acute/subacute phase.

Pooling all interventions in each post-stroke phase, UE rehabilitation interventions with a total of 3792 participants in the acute/subacute phase and 2753 participants in the chronic phase showed a mean difference of 5.04 and 2.14 in FMA-UE, respectively.

**Figure 2.**
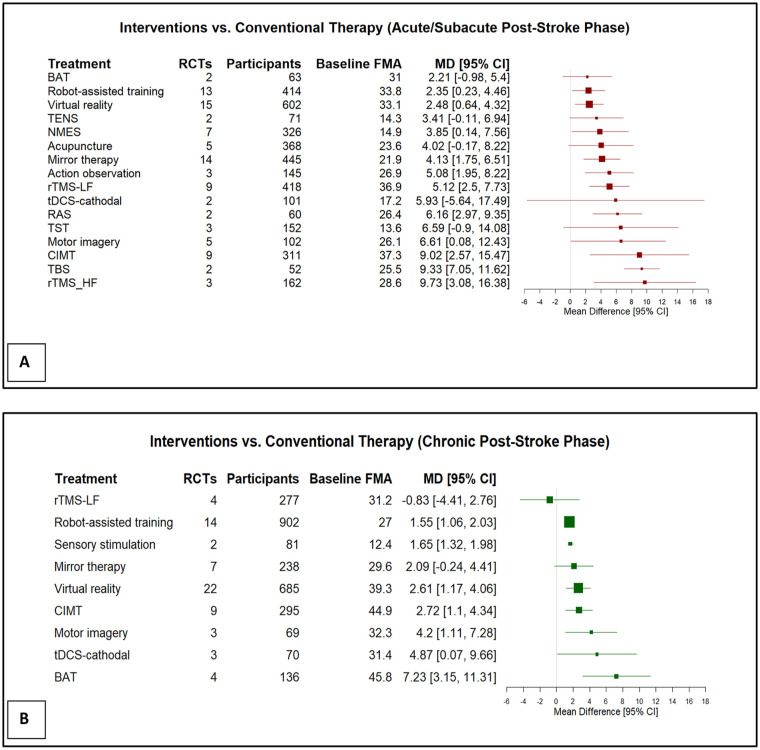
Forest plots of estimated mean differences (MD) (95% confidence intervals [CI]) comparing post-stroke UE rehabilitation interventions to conventional therapy in the (A) acute/subacute phase (≤6 months), and (B) chronic phase (>6 months).

## Discussion

This meta-analysis assessed the effect of 17 post-stroke UE rehabilitation interventions based on FMA-UE score improvements. Interventions studied in the acute/subacute post-stroke phase produced a markedly greater magnitude of improvement in FMA-UE scores than those studied in the chronic phase. This finding has important implications for patients, healthcare providers, and health systems, as well as future research. Across the 8 interventions assessed in both post-stroke phases, bilateral arm training was the only intervention with a significant and greater FMA-UE improvement in RCTs of chronic phase than those in acute/subacute phase.

The number of RCTs focused on post-stroke UE rehabilitation is notably robust, with our recent systematic review identifying 1276 RCTs. Of these, 53% of RCTs were conducted during the chronic phase, 24.8% in the subacute phase, and 19.3% during the acute phase.^21^ Despite the predominance of studies in the chronic phase, most patients who suffer a stroke receive their rehabilitation interventions in the acute and subacute phases.^28^ This discrepancy raises concerns regarding the generalizability of RCT findings from the chronic phase to clinical practice, which predominantly occurs in the acute and subacute (first 6 months) post-stroke period.

The current meta-analysis demonstrates a greater difference in FMA-UE scores in RCTs conducted during the acute and subacute phases compared to those in the chronic phase. This highlights the critical role of timing in stroke motor rehabilitation, as the initial months post-stroke are when the most rapid improvement typically occurs in the natural history of recovery, and neuroplasticity has the greatest impact.^29^ Supporting this, previous research has shown that an additional 20 hours of post-stroke rehabilitation significantly improves UE motor function in both the acute and subacute phases, while no such improvement is observed in the chronic phase.^30^ Further, large multi-site trials indicate non-significant effects of an additional 30 to 32 therapy hours in the chronic phase^31,32^ with significant improvement with an additional 300 hours.^33^ Ward et al.^34^ in an observational study of 224 stroke patients observed favorable outcomes from an intensive upper extremity rehabilitation program, which included 90 hours of therapy in the chronic phase. It would suggest that rehabilitation interventions studied in the chronic phase will be less likely to demonstrate a significant mean difference on FMA-UE than studies conducted in the acute/subacute phase for the same units of therapy. This is consistent with our finding that interventions studied in the chronic phase of stroke do not impact the Fugl-Meyer UE Assessment to the same degree as the same interventions conducted in the acute-subacute phase.

While meta-analyses do not consistently stratify outcome data by time post-stroke, reporting the effectiveness of interventions by timing is crucial for implementing appropriate rehabilitation strategies. Given the substantial differences in FMA-UE improvements between the acute/subacute and chronic phases, many of the post-stroke UE motor RCTs conducted in the chronic phase may undervalue the potential benefits of interventions. The lower levels of statistically significant improvements observed in chronic-phase RCTs are particularly concerning because rehabilitation is often limited during this timeframe. Consequently, these findings may restrict the generalizability of such trials to patients, clinicians, and health systems engaged in developing and funding effective rehabilitation programs, and may make a treatment appear less effective than similar studies conducted in the acute/subacute phase.

The potential for underestimating the impact of an intervention through research conducted in the chronic phase is further accentuated by the fact that the majority of outcomes in UE motor rehabilitation RCTs have been evaluated in the chronic phase post-stroke.^35^ Researchers may be inclined to conduct RCTs primarily in the chronic phase due to the ease of participant recruitment, as individuals who have completed their rehabilitation may show a higher willingness to enter clinical trials. Additionally, there are fewer mediating factors in the chronic phase, given that recovery typically plateaus after 6 months, and patients are less likely to receive other rehabilitation treatments concurrently. Inpatient rehabilitation environments may not always be conducive to rehabilitation trials that require active participation, as higher levels of cooperation and coordination are necessary, and research is often not prioritized.

This meta-analysis indicates that patients are likely to benefit most from studies focusing on interventions that optimize recovery during the acute and subacute phases, where meaningful improvements in UE function are more likely to occur, and this correlates with the time post stroke when stroke patients receive their rehabilitation. Conducting research in the chronic phase is of concern as those studies are likely to underestimate the impact of the treatment, at least on the FMA-UE, and studies conducted in the chronic phase have less generalizability, as discussed previously.

In addition to examining the magnitude of change, it is also important to consider the nature of that recovery and the degree to which improvements differ based on intervention type or baseline severity. Prior work has raised concerns about the assumption that similar FMA-UE gains reflect similar recovery processes. For example, Hawe et al.^36^ challenged the proportional recovery rule, demonstrating that improvements observed in many studies may be explained by mathematical coupling and ceiling effects, rather than a true biological process. Similarly, Scott et al.^37^ emphasized the need for more precise, kinematic-based metrics that compare stroke patients’ performance to healthy individuals, rather than relying solely on ordinal scales, in order to distinguish true motor recovery from compensatory strategies.

In this context, the notably higher mean differences (>9 points) observed for rTMS-HF, TBS, and mCIMT in the acute/subacute phase may suggest a subset of particularly effective therapies. These interventions share mechanisms such as targeted neurostimulation, which may enhance neuroplasticity when applied during the early post-stroke window.

However, it remains to what degree these effects represent genuine neurological recovery or are influenced by other factors, including baseline impairment. Without stratifying by initial FMA-UE score, one cannot determine whether patients with more severe deficits benefit equally from these interventions. Understanding how intervention type, timing, and initial severity interact is essential for developing personalized rehabilitation strategies. Future research should prioritize exploring these relationships to better guide clinical decision-making.

## Limitations

A protocol of this review was not previously registered. Only English studies were included in this review, which may have led to missing potentially relevant trials in other languages. The meta-analysis required the use of the FMA-UE subscale as an outcome measure for studies to be included, which may have reduced the number of interventions available for analysis.

Although the total FMA-UE (maximum of 66 points) was consistently used to ensure comparability across studies, variations in scoring methods or assessor training may have introduced heterogeneity. Most importantly, the use of the total FMA-UE score does not indicate which specific motor functions (e.g., proximal vs. distal upper limb) contributed to the observed improvements. This may limit the ability to assess whether changes were functionally meaningful or attributable to the specific interventions under study. Prior research has shown that improvements in certain FMA subscales may be more clinically relevant than changes in the overall score.^38^ Future studies should consider analyzing FMA-UE subscales (e.g., shoulder, wrist, hand, coordination) separately to better capture meaningful recovery patterns and intervention-specific effects.

Other risk of bias tools may be more suitable for assessing RoB in rehabilitation trials, given the nature of rehabilitation interventions. Since blinding therapists and participants is often impossible, many studies are classified as having a high risk of bias, even when all other domains indicate a low risk.

## Conclusions

The present study examined the effectiveness of UE rehabilitation interventions in RCTs relative to a conventional care or sham therapy control group during the acute/subacute phase and the chronic phase post-stroke, using FMA-UE as the outcome measure of interest. Our analysis showed that most interventions for post-stroke UE rehabilitation had beneficial effects on improving FMA-UE scores in both acute/subacute and chronic post-stroke phases. However, interventions studied in the acute/subacute phases, on average, showed greater improvements in the FMA-UE scores, compared to those conducted in the chronic phase, which fits with our understanding of the time-limited impacts of neuroplasticity and raises concerns about RCTs continuing to be conducted in the chronic phase and the generalizability of such research.

## Supplemental Material

sj-docx-1-nnr-10.1177_15459683251356975 – Supplemental material for Time Post-Stroke and Upper Extremity Stroke Motor Recovery Rehabilitation: A Meta-Analysis Supplemental material, sj-docx-1-nnr-10.1177_15459683251356975 for Time Post-Stroke and Upper Extremity Stroke Motor Recovery Rehabilitation: A Meta-Analysis by Sarvenaz Mehrabi, Cecilia Flores-Sandoval, Jamie L. Fleet, Sean P. Dukelow, Emma A. Bateman and Robert Teasell in Neurorehabilitation and Neural Repair

sj-pdf-2-nnr-10.1177_15459683251356975 – Supplemental material for Time Post-Stroke and Upper Extremity Stroke Motor Recovery Rehabilitation: A Meta-Analysis Supplemental material, sj-pdf-2-nnr-10.1177_15459683251356975 for Time Post-Stroke and Upper Extremity Stroke Motor Recovery Rehabilitation: A Meta-Analysis by Sarvenaz Mehrabi, Cecilia Flores-Sandoval, Jamie L. Fleet, Sean P. Dukelow, Emma A. Bateman and Robert Teasell in Neurorehabilitation and Neural Repair
