# Supplementary material for: Time Post-Stroke and Upper Extremity Stroke Motor Recovery Rehabilitation: A Meta-Analysis
Source: Neurorehabil Neural Repair. 2025 Jul 23;39(11):945–53. doi: 10.1177/15459683251356975 (PMC12531395; doi:10.1177/15459683251356975)
Supplement: sj-docx-1-nnr-10.1177_15459683251356975 – Supplemental material for Time Post-Stroke and Upper Extremity Stroke Motor Recovery Rehabilitation: A Meta-Analysis [file sj-docx-1-nnr-10.1177_15459683251356975.docx]

**Supplemental Material 1**

**Table S1.** List of included upper extremity motor rehabilitation interventions.

| **Intervention** | **Traditional description of the intervention** |
| --- | --- |
| Action observation (AO) | A cognitive and motor training technique where individuals watch someone else (a recorded video or a real demonstration), performing a specific action with the aim of learning or improving their own ability to perform the same action. The process involves activating brain regions associated with motor skills, particularly the mirror neuron system, which plays a role in motor learning, rehabilitation, and performance enhancement.  **Source**: Mulder, T. (2007). Motor imagery and action observation: cognitive tools for rehabilitation. *Journal of Neural Transmission*, 114(10), 1265-1278. <https://doi.org/10.1007/s00702-007-0763-z> |
| Acupuncture | A traditional Chinese medical practice that involves inserting needles into specific body points to stimulate nerves, muscles, and connective tissue and neurological and physiological responses that may promote recovery.  **Source:** Wu, P., Mills, E., Moher, D., & Seely, D. (2010). Acupuncture in poststroke rehabilitation: a systematic review and meta-analysis of randomized trials. Stroke, 41(4), e171-e179. <https://doi.org/10.1161/STROKEAHA.109.573618> |
| Bilateral arm training (BAT) | A rehabilitation approach, where both arms perform coordinated or simultaneous movements, often targeting the improvement of motor function in the affected arm. This method aims to activate both hemispheres of the brain to enhance neural plasticity and promote motor recovery in the impaired limb by engaging both arms in exercises, either symmetrically or asymmetrically.  **Source:** Whitall, J., McCombe Waller, S., Silver, K. H. C., & Macko, R. F. (2000). Repetitive bilateral arm training with rhythmic auditory cueing improves motor function in chronic hemiparetic stroke. Stroke, 31(10), 2390-2395. <https://doi.org/10.1161/01.STR.31.10.2390> |
| Cathodal transcranial direct stimulation (tDCS-C) | A non-invasive brain stimulation where a low electrical current is applied to the scalp using electrodes, with the cathode (negative electrode) placed over a targeted brain region to reduce neuronal cortical excitability.  **Source:** Nitsche, M. A., & Paulus, W. (2000). Excitability changes induced in the human motor cortex by weak transcranial direct current stimulation. The Journal of Physiology, 527(3), 633-639. <https://doi.org/10.1111/j.1469-7793.2000.t01-1-00633.x> |
| Constraint induced movement  therapy (CIMT) | A rehabilitation technique in which the unaffected limb is restrained (e.g., with a sling or mitt) to encourage the use of the affected limb for daily activities. There can be a modified version of CIMT, which modifies the therapy time of practicing shaping and transfer package activities. The patient typically uses a mitt to restrain the unaffected arm.  **Source**: Taub, E., Uswatte, G., & Pidikiti, R. (1999). Constraint-induced movement therapy: a new family of techniques with broad application to physical rehabilitation—a clinical review. *Journal of Rehabilitation Research and Development*, 36(3), 237-251. |
| Mental practice/motor imagery | It refers to the cognitive rehearsal of a specific task without actual movement, where individuals mentally simulate performing the task repetitively in the environment in which task performance would take place (usually by listening to an audiotape). This technique activates neural networks involved in motor control.  **Source**: Mulder, T. (2007). Motor imagery and action observation: cognitive tools for rehabilitation. *Journal of Neural Transmission*, 114(10), 1265-1278. <https://doi.org/10.1007/s00702-007-0763-z> |
| Mirror therapy | A mirror is placed between a patient's limbs so that the reflection of the unaffected limb appears in place of the affected limb, creating an illusion of two limbs as if they are both functioning normally. Participants are instructed to look at the reflection of the unaffected limb in the mirror perform bilateral symmetrical movements.  **Source**: Ramachandran, V. S., & Altschuler, E. L. (2009). The use of visual feedback, in particular mirror visual feedback, in restoring brain function. *Brain*, 132(7), 1693-1710. <https://doi.org/10.1093/brain/awp135> |
| Neuromuscular  electrical stimulation (NMES) | A technique that uses electrical impulses to evoke muscle contractions by stimulating the motor nerves. It can be delivered in repeated, timed cycles (Cyclic NMES), or be combined with voluntary muscle activity, detected by electromyography signals (EMG-triggered NMES). All modalities aim to improve muscle strength, prevent atrophy, enhance motor control, and promote recovery.  **Source:** Sheffler, L. R., & Chae, J. (2007). Neuromuscular electrical stimulation in neurorehabilitation. Muscle & Nerve, 35(5), 562-590. <https://doi.org/10.1002/mus.20758> |
| Rhythmic auditory stimulation (RAS) | A technique that uses rhythmic auditory cues, such as metronome beats or music, to facilitate the improvement of motor functions, particularly movement coordination and motor control.  **Source:** Thaut, M. H., Leins, A. K., Rice, R. R., Argstatter, H., Kenyon, G. P., McIntosh, G. C., & Fetter, M. (2007). Rhythmic auditory stimulation improves gait more than NDT/Bobath training in near-ambulatory patients early poststroke: a single-blind, randomized trial. Neurorehabilitation and Neural Repair, 21(5), 455-459. <https://doi.org/10.1177/1545968307300523> |
| Robot-assisted trainings | A group of trainings that use robotic devices to facilitate repetitive, controlled movements of the arm and hand. These devices assist or resist the patient’s movements to promote motor learning, muscle re-education, and neuroplasticity. Robot-assisted training may improve strength, range of motion, and motor function, particularly by allowing intensive and precise rehabilitation exercises.  **Source:** Lo, A. C., Guarino, P. D., Richards, L. G., Haselkorn, J. K., Wittenberg, G. F., Federman, D. G., & Volpe, B. T. (2010). Robot-assisted therapy for long-term upper-limb impairment after stroke. New England Journal of Medicine, 362(19), 1772-1783. <https://doi.org/10.1056/NEJMoa0911341> |
| Repetitive transcranial magnetic stimulation- high frequency (rTMS-HF) | A non-invasive brain stimulation technique that delivers rapid, repeated magnetic pulses to specific brain regions at high frequencies (typically ≥5 Hz) and increase cortical excitability. It is used to improve motor recovery by enhancing neural plasticity.  **Source:** Lefaucheur, J. P., Aleman, A., Baeken, C., Benninger, D. H., Brunelin, J., Di Lazzaro, V., & Ziemann, U. (2020). Evidence-based guidelines on the therapeutic use of repetitive transcranial magnetic stimulation (rTMS). Clinical Neurophysiology, 131(2), 474-528. <https://doi.org/10.1016/j.clinph.2019.11.002> |
| Repetitive transcranial magnetic stimulation- low frequency (rTMS-LF) | A non-invasive brain stimulation that delivers repeated magnetic pulses at low frequencies (typically 1 Hz or less) to a specific brain region. LF-rTMS is used to decrease cortical excitability and has therapeutic applications in post-stroke motor recovery by inhibiting overactive brain regions.  **Source**: Lefaucheur, J. P., Aleman, A., Baeken, C., Benninger, D. H., Brunelin, J., Di Lazzaro, V., & Ziemann, U. (2020). Evidence-based guidelines on the therapeutic use of repetitive transcranial magnetic stimulation (rTMS). *Clinical Neurophysiology*, 131(2), 474-528. <https://doi.org/10.1016/j.clinph.2019.11.002> |
| Sensory stimulation | Techniques that involve activating sensory pathways through stimuli, such as tactile (touch), exteroceptive (external stimuli like temperature or texture), and proprioceptive (awareness of body position and movement) inputs. These stimulations aim to enhance sensory perception, promote neuroplasticity, and improve motor function by facilitating the integration of sensory feedback, which is often impaired after a stroke.  **Source**: Schabrun, S. M., & Hillier, S. (2009). Evidence for the retraining of sensation after stroke: a systematic review. *Clinical Rehabilitation*, 23(1), 27-39. <https://doi.org/10.1177/0269215508098897> |
| Theta burst stimulation (TBS) | A form of rTMS that uses short bursts of magnetic pulses delivered at a frequency mimicking theta brain wave (5 Hz). Intermittent stimulation increases cortical excitability in the ipsilesional hemisphere.  **Source**: Huang, Y. Z., Edwards, M. J., Rounis, E., Bhatia, K. P., & Rothwell, J. C. (2005). Theta burst stimulation of the human motor cortex. *Neuron*, 45(2), 201-206. <https://doi.org/10.1016/j.neuron.2004.12.033> |
| Transcutaneous electrical nerve stimulation (TENS) | A non-invasive therapy that uses low-voltage electrical currents delivered through electrodes placed on the skin. The electrical stimulation modulates pain signals by activating sensory nerves. TENS may enhance motor recovery secondary to reducing pain, which can improve patient participation in rehabilitation exercises.  **Source**: Sluka, K. A., & Walsh, D. (2003). Transcutaneous electrical nerve stimulation: basic science mechanisms and clinical effectiveness. *The Journal of Pain*, 4(3), 109-121. <https://doi.org/10.1054/jpai.2003.434> |
| Task specific training (TST) | A rehabilitation approach that involves practicing functional tasks that are directly relevant to the individual's daily activities. It focuses on repetitive practice of specific movements or activities, such as reaching, grasping, or walking, to improve motor function and promote neuroplasticity. The goal is to enhance the patient's ability to perform everyday tasks by targeting the affected muscles and motor skills.  **Source:** French, B., Thomas, L. H., Leathley, M. J., Sutton, C. J., McAdam, J., Forster, A., & Langhorne, P. (2010). Repetitive task training for improving functional ability after stroke. Cochrane Database of Systematic Reviews, (7). <https://doi.org/10.1002/14651858.CD006073.pub2> |
| Virtual reality (VR) | A computer-generated simulation of a 3D environment that allows users to interact with it in a seemingly real or physical way using specialized equipment, such as VR headsets or gloves with sensors. It aims to simulate real-life activities, promote motor learning, and enhance cognitive or physical recovery. In the concept of UE motor rehabilitation, VR may either use for task-oriented activities that focused on gripping and handling of objects with upper extremity motion and stability or playing sports like boxing, bowling, tennis, golf, baseball, table tennis, basketball, cycling, Frisbee disk, and sword play that typically involve a variety of upper extremity motions including internal and external rotation of the shoulder, flexion and extension of the elbow, and pronation and supination of the forearm.  **Source**: Laver, K. E., Lange, B., George, S., Deutsch, J. E., Saposnik, G., & Crotty, M. (2017). Virtual reality for stroke rehabilitation. *Cochrane Database of Systematic Reviews*, (11). <https://doi.org/10.1002/14651858.CD008349.pub4> |

**Table S2.** List of randomized controlled trials included in the meta-analysis.

| **Author**  **Year**  **Country** | **Study title** | **Sample size**  **E: Experiment C: Control** | **Intervention group** | **Control group** | **Therapy intensity and duration** | **Time post-stroke** |
| --- | --- | --- | --- | --- | --- | --- |
| **Action Observation (AO)** | | | | | | |
| Zhu et al.^1^  2020  China (Mainland) | Visual feedback therapy for restoration of upper limb function of stroke patients. | E: 16  C: 15 | AO: Imitate a variety of activities of daily living movements with their affected arm after watching them on a video, included shoulder, elbow, wrist and dexterity range of motion practice, object grasping and manipulation. | Conventional Therapy:  Acute medical care and traditional rehabilitation training (i.e., exercise and occupational therapies) | 30min, 6d/wk, for 8wk AO  3-4hr/d, 6d/wk, for 8wk conventional therapy | Subacute |
| Fu et al.^2^  2017  China (Mainland) | Effects of action observation therapy on upper extremity function, daily activities and motion evoked potential in cerebral infarction patients. | E: 28 C: 25 | AO + Conventional therapy:  Participants watched upper limb motion videos and were informed to imitate the action in the video. Actions included shoulder, elbow, wrist, forearm and hand movements. | Conventional rehabilitation therapy and usual stroke care | 20min, 6d/wk, for 8wk, AO  Duration for conventional therapy is not specified. | Subacute |
| Zhu et al.^3^  2015  China (Mainland) | Effect of action observation therapy on daily activities and motor recovery in stroke patients. | E: 31  C: 30 | AO + Routine rehabilitation:  Participants were required to perform the same upper limb exercise movements after watching the specific actions on a video. | Routine rehabilitation treatment and nursing care | 30min, 6d/wk, for 8wk, AO  2-5hr/d, 6d/wk, for 8wk, routine rehabilitation | Acute |
| **Acupuncture** | | | | | | |
| Kim et al.^4^  2020  South Korea | Synergistic Effects of Scalp Acupuncture and Repetitive Transcranial Magnetic Stimulation on Cerebral Infarction: A Randomized Controlled Pilot Trial. | E1: 11  C: 12  Multi-arm | Scalp acupuncture (SA)+ Conventional Stroke Rehabilitation:  needles were horizontally inserted into the lesion site and two upper limb regions (MS6 and MS7). The needles were left in position for 20min. | Conventional Stroke Rehabilitation:  Practicing fine and gross motor movements, activities of daily living, and task-oriented exercises. | 20min SA + 30min rehabilitation,  2x/d, 5d/wk for 3wk | Acute |
| Wang et al.^5^  2020  China (Mainland) | Effects of acupuncture treatment on motor function in patients with subacute hemorrhagic stroke: A randomized controlled study. | E: 67  C: 66 | Acupuncture + Conventional treatment: Two main points (GV20, EXHN5) and two other optimum reaction points identified by motor-evoked potential tests were chosen. The limb points were selected based on hemiplegia phase. | Conventional treatment:  Standard routine pharmacologic stroke treatment. Physiotherapies for patients were carried out based on the hemiplegia phase. Program of exercises for atonic phase. | 6d/wk, for 4wks acupuncture  45 min/d, 6d/wk, for 4wks conventional therapy | Subacute |
| Zhang et al.^6^  2017  China (Mainland) | Neuronavigation-Assisted Aspiration and Electro-Acupuncture for Hypertensive Putaminal Hemorrhage: A Suitable Technique on Hemiplegia Rehabilitation. | E3: 30  C: 33  Multi-arm | E3: Electroacupuncture:  Scalp and body acupuncture on the paretic side, on 14 points based on traditional Chinese medicine. | Conventional therapy: Standard medication protocol and bedside rehabilitation program of functional exercises, including body movement and motor skills to avoid muscle atrophy and joint stiffness. | 30min, 2x/d, for 8wk either therapy | Acute |
| Hsieh et al.^7^  2007  China (Mainland) | Additional therapeutic effects of electroacupuncture in conjunction with conventional rehabilitation for patients with first-ever ischaemic stroke. | E: 30  C: 33 | Electroacupuncture + Conventional therapy:  Seven different acupuncture points on upper and lower limbs and neck. | Conventional therapy:  Conventional stroke rehabilitation, including physical and occupational therapy, with speech therapy if indicated. | 20min/session, 2d/wk, for 4wk electro-acupuncture.  Duration of conventional therapy is not specified. | Acute |
| Alexander et al.^8^  2004  United States | Effects of acupuncture treatment on poststroke motor recovery and physical function: a pilot study. | E: 14 C: 15 | Acupuncture+ Conventional rehabilitation:  A standardized approach included manual needle insertion to the hemiparetic limb. The acupuncturist could select specific sites based on patient symptoms. | Conventional rehabilitation:  Conventional stroke rehabilitation care, including physical, occupational, and/or speech therapy for the duration of the inpatient stay. | 30min/d, 7d/wk, for 2wks Acupuncture  3hr/d, 6d/wk, for 2wk  rehabilitation | Acute |
| **Bilateral Arm Training (BAT)** | | | | | | |
| Lee et al.^9^  2019  South Korea | The effect of bilateral upper limb training on recovery of upper limb function in patients with acute Stroke. | E: 15 C: 15 | BAT: Included wiping, drinking, moving blocks to boxes. | Conventional Rehabilitation of the upper limb | 30min/d, 5d/wk, for 4wk either therapy | Subacute |
| Lee et al.^10^  2017  South Korea | Effectiveness of bilateral arm training for improving extremity function and activities of daily living performance in hemiplegic patients. | E: 15 C: 15 | BAT + General OT:  BAT included dishwashing, making coffee, typing, cutting fruit, and folding laundry. | General OT:  Included stretching, posture, resistance movements, and fine motor training. | 1hr, 5d/wk, for 8wk either therapy | Chronic |
| Lin et al.^11^  2015  Taiwan, China | Effects of computer-aided Interlimb force coupling training on paretic hand and arm motor control following chronic stroke: A randomized controlled trial. | E:16  C:17 | BAT: Novel bimanual training program including bilateral isometric handgrip force training. | Routine Rehabilitation:  Included strengthening, stretching, functional tasks, coordination, and weight bearing training of the hemiparetic upper limb. | 30min/d, 3d/wk for 4wk BAT  Routine rehabilitation in duration of the study period (4wk). | Chronic |
| Lin et al.^12^  2010  Taiwan, China | The effects of bilateral arm training on motor control and functional performance in chronic stroke: a randomized controlled study. | E: 16 C: 17 | BAT: Simultaneous movement and symmetrical functional tasks including lifting two cups, stack two checkers, pick up two small, dried beans, fold two towels, turn two large screws, manipulate two coins simultaneously. | Conventional Rehabilitation:  Included trunk-arm control, weight bearing, fine motor task practice, and compensatory strategies for activities of daily living. | 2hr/d, 5d/wk for 3wk either therapy | Chronic |
| Lin et al.^13^  2009  Taiwan, China | Effects of constraint-induced therapy versus bilateral arm training on motor performance, daily functions, and quality of life in stroke survivors. | E2: 20 C: 20  Multi-arm | BAT: Including functional tasks in symmetric or alternating patterns, such as lifting two cups, two pegs, reaching forward, and grasping and releasing two towels. | Conventional care:  Included training for hand function, coordination, balance, limb movement, compensatory practice and functional tasks. | 2hr/d, 5d/wk, for 3wk either therapy | Chronic |
| Desrosiers et al.^14^  2005  Canada | Effectiveness of unilateral and symmetrical bilateral task training for arm during the subacute phase after stroke: a randomized controlled trial. | E: 17 C: 16 | BAT + Conventional therapy:  BAT included symmetrical bilateral tasks such as wringing a garment, rolling a cylinder; asymmetrical tasks like making coffee. | Conventional therapy (OT and PT):  Consisted of functional activities and passive movements. | 45 min/d, 4d/wk, for 5wk either therapy. | Subacute |
| **Cathodal Transcranial Direct Current Stimulation (tDCS)** | | | | | | |
| Alisar et al.^15^  2020  Turkey | Effects of bihemispheric transcranial direct current stimulation on upper extremity function in stroke patients: a randomized double-blind sham-controlled study. | E: 16  C: 16 | Cathodal tDCS + Conventional Therapy: tDCS was applied using a double channelled direct current stimulator. The current was applied using 5.5 cm x 4 cm (22 cm^2^) rectangular electrodes. | Sham tDCS + conventional therapy (OT and PT): Included neurofaciliation techniques, range of motion, progressive resistance and strengthening exercises | 30min/d, 5d/wk, for 3wk stimulation  60-120min/d, 5d/wk, for 3wk PT and OT | Chronic |
| Lee & Lee^16^  2015  South Korea | Effects of adjustment of transcranial direct current stimulation on motor function of the upper extremity in stroke patients | E: 16 C: 16 | Cathodal tDCS + PT: stimulation to the cerebral cortex motor area. | PT: Components not specified | 5x/wk for 4wk | Chronic |
| Fusco et al.^17^  2014  Italy | The ineffective role of cathodal tDCS in enhancing the functional motor outcomes in early phase of stroke rehabilitation: an experimental trial. | E: 5 C: 6 | Cathodal tDCS + Conventional Rehabilitation: Stimulation of the primary motor cortex in the contra-lesional hemisphere at a current density of 0.043mA/cm^2^. | Sham stimulation + Conventional Rehabilitation: Included motor rehabilitation of the upper and lower limb | 10min/d, 5d/wk, for 2wk Stimulation  45min, 2x/d, 5d/wk, for 2wk Rehabilitation | Acute |
| Wu et al.^18^  2013  China (Mainland) | Effects on decreasing upper-limb poststroke muscle tone using transcranial direct current stimulation a randomized sham controlled study | E: 45 C: 45 | Cathodal tDCS + Conventional PT: Cathodal stimulation over C3 or C4 at 1.2 mA. | Sham stimulation + Conventional PT:  PT included stretching, positioning, and movement training. | 20min/d, 5d/wk, for 4wks stimulation  60min/d, 5d/wk, for 4wks PT | Subacute |
| Nair et al.^19^  2011  United States | Optimizing recovery potential through simultaneous occupational therapy and Non-invasive brain-stimulation using tDCS | E: 7 C: 7 | Cathodal tDCS + OT:  Stimulation over the contra-lesional motor region (either C3 or C4 of the 10–20 EEG system) and a reference electrode over the contralateral supraorbital region. | Sham stimulation + OT:  OT included shoulder abduction, external rotation, elbow extension, and forearm pronation. | 30min/d, 5d/wk, for 1wk stimulation  60min/d, 5d/wk, for 1wk OT | Chronic |
| **Constraint-Induced Movement Therapy (CIMT)** | | | | | | |
| Abdullahi^20^  2018  Nigeria | Effects of Number of Repetitions and Number of Hours of Shaping Practice during Constraint-Induced Movement Therapy: A Randomized Controlled Trial | E1: 13  C: 12  Multi-arm | mCIMT: The shaping  practice performed included activities such as picking up a cup from the table, writing letters or drawing a circle, transferring an object, brushing the teeth, putting and removing shoes, etc. | Traditional Therapy: Consisting of passive movement, therapeutic positioning, and weight bearing on the affected limb | 3hr, 5x/wk for 4wk | Acute |
| Yadav et al.^21^  2016  India | Efficacy of modified constraint induced movement therapy in the treatment of hemiparetic upper limb in stroke patients: A randomized controlled trial | E: 30 C: 30 | mCIMT + Conventional rehabilitation: Focused functional tasks like reaching forward to hold a glass and drinking from it, picking up a comb and combing hair, turning on and off a light switch, buttoning and unbuttoning of clothes, writing with a pen. The patient’s unaffected hand and wrist was covered with a mitt | Conventional rehabilitation including activities of daily living training, stretching, range of motion and strengthening exercises, endurance training, gait training, orthosis and education | 5h/d, 5d/wk mCIMT  3h/d, 5d/wk for 4wk Conventional Rehabilitation | Chronic |
| Kwakkel et al.^22^  2016  Netherlands | Effects of Unilateral Upper Limb Training in Two Distinct Prognostic Groups Early After Stroke | E1: 29  C: 29  Multi-arm | mCIMT: Patients received supervised intensive graded practice focused on improving task-specific use of the paretic arm and hand. Patients were instructed to wear a padded safety mitt. | Usual care: Usual upper limb therapy in both strata consisted of exercise therapy based on recommendations from current Dutch guidelines18 applied face-to-face by a physical therapist or occupational therapist for 30 minutes per working day executed for 3 consecutive weeks. | 60min/session, 5d/wk, 3wks training while wearing the pads for 3hr/d, 5d/wk.  30min, 5d/wk, 3wk, Usual care | Acute |
| Thrane et al.^23^  2015  Norway | Efficacy of Constraint-Induced Movement Therapy in Early Stroke Rehabilitation: A Randomized Controlled Multisite Trial | E: 24  C: 23 | CIMT: Focused on repetitive task-oriented training, shaping and task practice, and adherence-enhancing behavioral strategies. Patients were encouraged to wear a constraining mitt on the more affected arm. | Usual Care: Components unspecified | 3h, 1/d for 10d | Acute |
| El-Helow et al.^24^  2015  Egypt | Efficacy of modified constraint-induced movement therapy in acute stroke | E: 30 C: 30 | mCIMT: Consisted of movement restriction and shaping. Restriction of movement of the unaffected extremity was achieved by asking the patient to wear a mitten on the un-affected hand during the treatment period. | Conventional Rehabilitation: Including traditional positioning, occupational therapy, compensatory techniques, strengthening and range of motion exercises | 6h/d, 2wk mCIMT  2h/d, 5d/wk for 2wk Conventional rehabilitation | Subacute |
| Yoon et al.^25^  2014  South Korea | Effect of Constraint-Induced Movement Therapy and Mirror Therapy for Patients With Subacute Stroke | E2: 9  C: 9  Multi-arm | CIMT + Conventional OT: Patients wore a specially designed orthosis to suppress the motion of the unaffected upper extremity. | Conventional OT: including self-exercise program and routine rehabilitation in hospital | 6h/d CIMT + 40min/d Conventional OT, 5d/wk for 2wk  40 min/d + 30 min self-exercise | Subacute |
| vanDelden et al.^26^  2013  Netherlands | Unilateral versus bilateral upper limb training after stroke: the Upper Limb Training After Stroke clinical trial. | E1: 21  C: 16  Multi-arm | mCIMT: Involved repetitive task practices and shaping of the desired movements, with an emphasis on the increase  of control of wrist and finger extensors. Patients wore a mitt on the nonparetic hand. | Dose-matched Conventional Treatment: Based on existing guidelines for upper limb rehabilitation | 1h, 3d/wk for 6wk | Subacute |
| Singh & Pradhan^27^  2013  India | Study to assess the effectiveness of modified constraint-induced movement therapy in stroke subjects: A randomized controlled trial. | E: 20 C: 20 | mCIMT: A mitt was used to restrain the unaffected arm which prevented the use of unaffected limb. It was made of cotton material which was extending till the forearm. Patients were encouraged to wear a mitt during treatment and post treatment except for activities like toileting, washing etc. | Standard PT: Treatment included compensatory technique for activities of daily living activity, strength, and range of motion and traditional positioning for affected arm. | 120min of shaping, 5x/wk for 2wk.  Constraint for 10hr, 5x/wk, for 3wk, 5x/wk, for 2wk. | Acute |
| Wu et al.^28^  2012  Taiwan, China | Pilot trial of distributed  constraint induced therapy  with trunk restraint to improve poststroke reach to grasp and trunk kinematics | E2: 15  C: 15  Multi-arm | Distributed CIMT: The functional tasks included flipping pages of magazines and picking up coins. The shaping skills involved individualized task selection, graded task difficulty, verbal feedback, prompting, physical assistance with movements, and modeling | Usual Care: Including neurodevelopmental techniques, strength, muscle tone, range of motion, body posture and functional task performance | 2h/d, 5d/wk, 3 wk | Chronic |
| Lin et al.^29^  2010  Taiwan, China | Constraint-induced therapy versus control intervention in patients with stroke: a functional magnetic resonance imaging study. | E: 5  C: 8 | Distributed CIMT: Involved intensive training of the affected limb based on a task-oriented approach that emphasized repetitive practice of functional activities and behavioral shaping (e.g., moving a cup, picking up coins, combing hair, writing). The shaping procedure involved individualized task selection, graduated task difficulty, verbal feedback, prompting, physically assisting with movements, and modeling. | Routine Therapy including neurodevelopmental techniques, stretching, weight bearing and fine motor tasks | 2h, 5d/wk for 3wk | Chronic |
| Lin et al.^13^  2009  Taiwan, China | Effects of constraint-induced therapy versus bilateral arm training on motor performance, daily functions, and quality of life in stroke survivors | E1: 20 C: 20  Multi-arm | CIMT: Consisting of two hours of shaping (tasks included reaching forward, picking up coins, picking up a utensil), and six hours of constraint. | Conventional Therapy: Focusing on hand function, coordination, balance and movements of the affected limb. | 120min of shaping, 5x/wk for 3wk  Constraint for 6hr, 5x/wk for 3wk. | Chronic |
| Lin et al.^30^  2009  Taiwan, China | Constraint-induced therapy versus dose-matched control intervention to improve motor ability, basic/extended daily functions, and quality of life in stroke. | E: 16 C: 16 | CIMT: Focused on functional training of the affected limb. Shaping, adaptive, and repetitive practice of functional tasks were used during training sessions (e.g., dialing a phone number, reaching forward to move a jar, picking up a cup and drinking from it) | Conventional Rehabilitation: Focused on  neurodevelopmental techniques emphasizing functional task  practice, when possible, as well as weight bearing by the affected  limb and fine motor dexterity activities | 120min of shaping, 5x/wk for 3wk.  Constraint for 5hr, 5x/wk for 3 wk. | Chronic |
| Page et al.^31^  2008  United States | Modified constraint-induced therapy in chronic stroke: results of a single-blinded randomized controlled trial. | E: 13 C1: 12/10  Multi-arm | mCIMT: Shaping was applied with 2 or 3 upper-limb activities (e.g., writing, using a fork). Affected arms were restrained using a cotton hemi-sling, while their hands were placed in mesh, polystyrene-filled mitts with  Velcro straps around the wrist. | Time-Matched Rehabilitation: Focused on functional tasks, stretching, shoulder extension, adduction and rotation, finger and wrist flexion. Use of compensatory techniques with unaffected side as needed. | 30min of shaping, 3x/wk for 10wk.  Constraint for 5hr, 5x/wk for 10wk. | Chronic |
| Lin et al.^32^  2008  Taiwan, China | A randomized controlled trial of constraint-induced movement therapy after stroke. | E: 12  C: 10 | CIMT: Restraint of the less affected limb combined with intensive training of the affected limb | Conventional Therapy: Neurodevelopmental techniques including weight bearing and dexterity tasks | 2h, 5x/wk for 3wk | Chronic |
| Boake et al.^33^  2007  United States | Constraint-induced movement therapy during early stroke rehabilitation. | E: 9 C: 7 | mCIMT: Task movements included reaching, grasping, lifting, and placing. | Traditional therapy: Consisting of activities intended to improve strength, muscle tone and range of motion. | 180min of shaping, 6x/wk for 2wk  Constraint for 90% of waking hr | Subacute |
| Wu et al.^34^  2007  Taiwan, China | Kinematic and clinical analyses of upper-extremity movements after constraint-induced movement therapy in patients with stroke: a randomized controlled trial. | E: 24 C: 23 | CIMT: Typical training activities involved the use of the more affected UE and were similar to those performed daily (e.g., reaching forward to move a jar from one place to another, picking up a cup and drinking from it, picking up a hairbrush and combing hair, cleaning the window). | Conventional therapy: Including functional task practice, stretching, weight bearing and fine motor tasks. | 120min of shaping, 5x/wk for 3wk  Constraint for 6h. | Chronic |
| Wu et al.^35^  2007  Taiwan, China | A randomized controlled trial of modified constraint-induced movement therapy for elderly stroke survivors: changes in motor impairment, daily functioning, and quality of life. | E: 13  C: 13 | mCIMT: Restraint of the unaffected limb combined with intensive training of the affected limb | Conventional Rehabilitation: Including neurodevelopmental techniques, stretching, weight bearing and motor dexterity | 2hr, 5x/wk for 3wk | Chronic |
| Page et al.^36^  2005  United States | Modified constraint-induced therapy in acute stroke: a randomized controlled pilot study | E: 5 C: 5 | mCIMT: The hands and wrists were restrained using polystyrene-filled mitts with Velcro straps around the wrist. | Traditional rehabilitation: Stretching, weight bearing, fine motor tasks, and activities of daily living. | 120min of shaping, 3x/wk, for 10wk  Constraint for 5h, 3x/wk for 10wk | Acute |
| **Repetitive Transcranial Magnetic Stimulation (rTMS)- High Frequency** | | | | | | |
| Mahdy Ibrahim et al.^37^  2020  Egypt | Effect of high frequency repetitive transcranial magnetic stimulation of the contralesional dorsal premotor cortex on recovery from post-stroke severe motor impairment. | E2: 20  C: 20 | rTMS (5Hz): rTMS was delivered using The  Magstim Rapid system with a 70 mm figure-of-eight uncoated flat coil. Each train of pulses consisted of five single pulses with an  interstimulus interval of 100ms, thus lasting for 400ms. | Sham rTMS: Sham  rTMS was delivered using a sham  Magstim coil that delivers only 5% of the  stimulator output (Magstim Company,  UK). | 10 sessions | Subacute |
| Guan et al.^38^  2017  China | Effectiveness of repetitive transcranial magnetic stimulation (rTMS) after acute stroke: A one-year longitudinal randomized trial. | E: 19  C: 18 | rTMS (5Hz): rTMS was conducted using a Medtronic MagPro type magnetic stimulation device (Medtronic,  Minneapolis, MN, USA) and a figure-eight coil (MC-B70, Medtronic). The treatment involved 50 trains of 20 pulses with 2-second intertrain intervals daily. | Sham rTMS: Coils were placed perpendicular to the scalp | 10d | Acute |
| Li et al.^39^  2016  China | Effects of different frequencies of repetitive transcranial magnetic stimulation on the recovery of upper limb motor dysfunction in patients with subacute cerebral infarction. | E2: 43  C: 42  Multi-arm | rTMS (10Hz) + Conventional Rehabilitation: rTMS was  conducted using a magnetic stimulator (YRD  Company, Wuhan, China), connected to a prototype round coil; 125 mm in diameter and a peak magnetic field of 3.0 T. | Sham rTMS + Conventional Rehabilitation: Including task-oriented training, motor training, and range of motion exercises. The sham group used a false  coil (only noise, but no substantial stimulus effect). | 20min/d, 5d/wk for 2wk rTMS  40min, 5x/wk, for 2 wk Conventional rehabilitation | Subacute |
| **Repetitive Transcranial Magnetic Stimulation (rTMS)- Low Frequency** | | | | | | |
| Kim et al.^4^  2020  South Korea | Synergistic Effects of Scalp Acupuncture and Repetitive Transcranial Magnetic Stimulation on Cerebral Infarction: A Randomized Controlled Pilot Trial. | E2: 18  C: 12  Multi-arm | rTMS (1Hz) + Conventional Stroke Rehabilitation Therapy: A 70 mm figure-8 coil and a Magstim Rapid stimulator (Magstim Co., Dyfed, UK) were used to deliver 1 Hz of rTMS to the skull of the contralesional hemisphere at the site that elicited the largest MEPs in the first dorsal interosseous muscle of the unaffected upper limb. One rTMS session consisted of 1200 pulses. | Conventional Stroke Rehabilitation Therapy: Focused on practicing fine and gross motor movements, activities of daily living, task-oriented therapeutic exercises, and muscular electrical stimulation therapy as needed, swallowing and dysarthria training was also provided. | 20min rTMS  30min, 2x/d, for 3wk Conventional Rehabilitation | Acute |
| Sharma et al.^40^  2020  India | Efficacy of Low-Frequency Repetitive Transcranial Magnetic Stimulation in Ischemic Stroke: A Double-Blind Randomized Controlled Trial | E: 47  C: 49 | rTMS (1Hz) + Conventional PT: Low frequency rTMS was performed using Magstim Rapid stimulator equipped with air cooled figure of 8 coil (70 mm). | Sham rTMS + Conventional PT: Sham rTMS pulses were administered using the same stimulation parameters. Components of PT not specified. | 10 sessions/2wk rTMS  45min/d PT | Acute |
| El-Tamawy et al.^41^  2019  Egypt | Effect of Repetitive Transcranial Magnetic Stimulation on Cortical and Motor Outcomes Post Stroke: A Randomized Controlled Trial | E: 20  C: 20 | rTMS (1Hz) + PT: The Magstim  Rapid2 magnetic stimulator system (Model P/N 3576-23-09, Magstim Company, USA) was used to deliver 1-Hz stimulation at 90% of the Active Motor Threshold (AMT) to the “hot spot” of the primary cerebral cortex (M1) in the contralesional hemisphere via a 70-mm figure- 8 coil. | Upper Limb PT: Components not specified. | 20min/d, for 2wk | Subacute |
| Long et al.^42^  2018  China (Mainland) | Effects of combining high- and low-frequency repetitive transcranial magnetic stimulation on upper limb hemiparesis in the early phase of stroke | E2: 21  C: 20  Multi-arm | rTMS (1Hz) + PT/OT: The rTMS was delivered using a 125-mm circular coil connected to a CCY-1  stimulator (YIRUIDE Medical Equipment Company,  Wuhan, China). | Sham rTMS + PT/OT: The sham group received sham stimulation at the  same site. PT consisted of activities to improve transfers, posture, balance, strength, and coordination and to provide sensory stimulation. OT included functional tasks and activities of daily living. | 15 sessions  30min/d, 6d/wk PT + 60min/d, 6d/wk OT | Acute |
| Harvey et al.^43^  2018  United States | Randomized sham-controlled trial of navigated repetitive transcranial magnetic stimulation for motor recovery in stroke: the NICHE trial. | E: 132  C: 37 | rTMS (1Hz) + Task-oriented rehabilitation therapy: rTMS was delivered using 1 Hz frequency to the cortical target on non-injured hemisphere. | Sham rTMS + Task-oriented rehabilitation therapy: Therapy sessions included pre-functional upper limb therapy focusing on basic shoulder and elbow mobilization. The  sham coil provided no stimulation. | 15min, 3x/wk, 6wk rTMS  60min task-Oriented rehabilitation | Chronic |
| Yang et al.^44^  2017  China (Mainland) | Effects of repetitive transcranial magnetic stimulation combined with sensory cueing on unilateral neglect in subacute patients with right hemispheric stroke: A randomized controlled study. | E2: 20  C: 20  Multi-arm | rTMS (1Hz): Inhibitory rTMS at 1 Hz was applied over P5 (using the international EEG 10/20 system) of the contralesional hemisphere at an intensity of 90% of the individual’s resting motor threshold. The stimulus was delivered at 900 pulses/session. | Conventional Rehabilitation: including PT and OT | 1x/d for 2wk rTMS  30 sessions, 45min 5x/wk Conventional Rehabilitation | Subacute |
| Tosun et al.^45^  2017  Turkey | Effects of Low-Frequency repetitive transcranial magnetic stimulation and neuromuscular electrical stimulation on upper extremity motor recovery in the early period after stroke: A preliminary study. | E1: 9  C: 9  Multi-arm | rTMS (1Hz) + Standardized PT and OT: A TAMAS device (Remed, Korea) with a figure-8 coil was used for rTMS. A frequency of 1 Hz, 1200 pulses with an intensity of 90% of resting motor threshold was delivered to the unaffected hemisphere | Standardized PT and OT: Including activities to improve strength, flexibility, transfers, posture, balance, coordination and activities of daily living. | 10 sessions, 5d/wk for 2wk rTMS  20 sessions PT-OT, 5d/wk for 4wk | Subacute |
| Askin et al.^46^  2017  Turkey | Effects of low-frequency repetitive transcranial magnetic stimulation on upper extremity motor recovery and functional outcomes in chronic stroke patients: A randomized controlled trial | E: 20  C: 20 | rTMS (1Hz) + Conventional PT: A TAMAS device (Remed, Daejeon, Korea) with a figure-of-eight coil was used for rTMS. A frequency of 1 Hz, 1200 pulses with an  intensity of 90% of RMT was delivered to the unaffected  hemisphere. | Conventional PT: The PT program included activities to improve strength, flexibility, transfers, posture, balance, coordination, and activities of daily living, mainly focusing on upper limb movements. | 10 sessions, 20minrTMS, 5d/wk for 4wk  20 sessions PT, 5d/wk for 4wk | Chronic |
| Li et al.^39^  2016  China (Mainland) | Effects of different frequencies of repetitive transcranial magnetic stimulation on the recovery of upper limb motor dysfunction in patients with subacute cerebral infarction. | E1: 42  C: 42  Multi-arm | rTMS (1Hz) + Conventional Rehabilitation: rTMS was  conducted using a magnetic stimulator (YRD Company, Wuhan, China), connected to a prototype round coil; 125 mm in diameter and a peak magnetic field of 3.0 T. | Sham rTMS + Conventional Rehabilitation: Including task-oriented training, motor training, and range of motion exercises. The sham group used a false coil (only noise, but no substantial stimulus effect). | 20min/d, 5d/wk for 2wk rTMS  40min, 5x/wk, for 2 wk Conventional rehabilitation | Subacute |
| Hosomi et al.^47^  2016  Japan | Daily Repetitive Transcranial Magnetic Stimulation for Poststroke Upper Limb Paresis in the Subacute Period | E: 18  C: 21 | rTMS (5Hz) + Regular Rehabilitation: rTMS was applied using a figure-8 coil (MC B-70; Medtronic Functional Diagnostics A/S, Skovlunde, Denmark; or no. 9925-00; Magstim Co Ltd, Whitland, United Kingdom) connected to a magnetic stimulator (MagPro, Medtronic Functional Diagnostics A/S; or Magstim Rapid, Magstim), which provided repetitive biphasic pulses. | Sham stimulation + Regular Rehabilitation: OT including gross motor training of hand dexterity, coordination with both hands and activities of daily living. | 1x/d, 5d/wk, for 2wk rTMS  20min, 8 sessions/d Regular rehabilitation | Subacute |
| Barros Galvao et al.^48^  2014  Brazil | Efficacy of coupling repetitive transcranial magnetic stimulation and physical therapy to reduce upper-limb spasticity in patients with stroke: a randomized controlled trial. | E: 10  C: 10 | rTMS (1Hz) + PT: The rTMS of the motor cortex was performed with a 70-mm figure-8 coil attached to a magnetic stimulator. | C: Sham + PT: Including activities for strength, flexibility, transfers, posture, balance, coordination and sensory stimulation, upper limb movements. For sham rTMS, no current was induced in the brain. | 10 sessions, 3d/wk rTMS  30min, 3d/wk PT | Chronic |
| Etoh et al.^49^  2013  Japan | Effects of repetitive transcranial magnetic stimulation on repetitive facilitation exercises of the hemiplegic hand in chronic stroke patients. | E: 9  C: 9 | rTMS: (1Hz): rTMS was applied using a 70-mm figure-of-eight coil and a Magstim Rapid stimulator (Magstim Co., Dyfed, UK). rTMS was applied for 4min, and comprised 240 pulses over the motor cortex of the unaffected hemisphere at a frequency of 1 Hz and a stimulus intensity of 90% of  the resting motor threshold. | Sham rTMS: 70-mm figure-of-eight coil and a Magstim Rapid stimulator (Magstim Co., Dyfed, UK). | 1x/d, 5d/wk for 2wk rTMS  1/d, 5d/wk for 2wk Sham | Chronic |
| Seniow et al.^50^  2012  Poland | Transcranial magnetic stimulation combined with physiotherapy in rehabilitation of poststroke hemiparesis: a randomized, double-blind, placebo-controlled study. | E: 20  C: 20 | rTMS (1Hz) + PT: Repetitive transcranial magnetic stimulation was performed by a single investigator (KW), using a Magstim Rapid Stimulator (Magstim Company, Whitland, UK) equipped with an air-cooled figure-of-eight coil (each loop 70 mm in diameter). | Sham rTMS + PT: Sham stimulation was performed with a coil that imitates the sound of a real TMS coil. All participants received individual PT in  accordance with the neurodevelopmental treatment/Bobath  concept. | 30min rTMS, 5d/wk for 3wk  45min PT, 5d/wk for 3wk | Subacute |
| **Mirror Therapy** | | | | | | |
| Chinnavan et al.^51^  2020  Malaysia | Effectiveness of Mirror Therapy on Upper Limb Motor Functions Among Hemiplegic Patients | E: 13  C: 12 | Mirror therapy + Conventional Rehabilitation: The therapy in the mirror group involved reaching, grasping and dexterity. | Conventional Therapy: The therapy in the conventional group involved mobilization, reaching, grasping and dexterity. | 45min, 3d/wk for 6wk | Chronic |
| Madhoun et al.^52^  2020  China (Mainland) | Task-based mirror therapy enhances the upper limb motor function in subacute stroke patients: a randomized control trial | E: 15  C: 15 | Task-based Mirror Therapy + Conventional therapy (if needed): Activities included elbow flexion, extension, ulnar and radial deviation, flexion and extension of the wrist, flexion and extension for the fingers, abduction, and adduction for all the fingers. Activities were conducted using various objects such as a spongy ball, a bottle of water, a duster, chopstick, a cup, cubes, wooden blocks, etc. | Sham + OT (if needed): The control group received OT without a mirror in addition to conventional therapy if the patients required. | 25min, 7d/wk for 4wk | Subacute |
| Guo et al.^53^  2019  China (Mainland) | Clinical study of combined mirror and extracorporeal shock wave therapy on upper limb spasticity in poststroke patients. International journal of rehabilitation research. | E1: 30  C: 30  Multi-arm | Mirror Therapy + Conventional Therapy: The affected hand was placed behind the mirror so that it could not be seen, and the unaffected hand was placed in the reflecting side of the mirror. Patients were asked to move their wrist while simultaneously observing the reflection of the unaffected hand. | Conventional Therapy: Including conventional exercise therapy, OT, and neurodevelopmental facilitation techniques | 20m/d, 5x/wk, for 4wk Mirror therapy  30min/d, 5x/wk, for 4wk Conventional therapy | Subacute |
| Bai et al.^54^  2019  China (Mainland) | Comparison between movement-based and task-based mirror therapies on improving upper limb functions in patients with stroke: A pilot randomized controlled trial. | E1: 12  E2: 11  C: 11  Multi-arm | E1: Movement-based mirror therapy + Conventional Therapy: Participants performed simple movements with the affected upper limb, such as finger tapping, griping and releasing, wrist, forearm, and elbow movements.  E2: Task-based mirror therapy + Conventional Therapy: Six tasks were performed with the affected hand, including transferring small cubes, placing pegs in holes and taking them out, turning over paper cards, placing steel needles in holes, stacking blocks, and putting cups on a shelf. | Conventional Therapy: Patients underwent multi-disciplinary rehabilitation training, including customary PT and OT. PT focused on the patients’ lower limb motor function and ambulation. Physiotherapists applied muscle stretching before active motor training. | 30min 5x/wk for 4wk Mirror therapy  1-2hr/d  Conventional therapy | Subacute |
| Antoniotti et al.^55^  2019  Italy | No evidence of effectiveness of mirror therapy early after stroke: an assessor-blinded randomized controlled trial | E: 16  C: 19 | Mirror therapy + Conventional therapy: Patients were asked to move their sound arm while looking the mirror reflective surface. | Sham + Conventional care: Conventional rehabilitation consisted of PT and OT, according to the physician prescription. In Sham mirror therapy, the mirror surface was opaque. | 30min 5d/wk for 4wk Mirror therapy  90min minimum, 5d/wk, for 4wk Conventional care | Acute |
| Ding et al.^56^  2019  China (Mainland) | Camera-Based Mirror Visual Input for Priming Promotes Motor Recovery, Daily Function, and Brain Network Segregation in Subacute Stroke Patients. | E: 9  C: 10 | Camera Mirror Visual Feedback (MFV): A customized camMVF was employed to present MVF instead of a real mirror. Two cameras were mounted on the lateral edge of the prism to capture the movements of hands, wrists, and forearms. During the training, the pictures of unaffected hand and its mirror image were shown on the screen in front of the patients. | Conventional Therapy: Included repetitive passive/active motor tasks and task-based motor training (dosage-equivalent) | 1.5hrs, 5d/wk for 4wk | Subacute |
| Ding et al.^57^  2018  China | Camera-based mirror visual feedback: potential to improve motor preparation in stroke patients. | Subacute subgroup:  E: 29  C: 29  Chronic subgroup:  E: 12  C: 12 | Camera-based Mirror Visual Feedback (MVF), mirror box: Two cameras were mounted on the top of the mirror box to capture the movements of the less-affected hand of stroke patients, the picture of the less-affected hand and its mirrored image were shown on the screen, which was superimposed just above the real hands | Conventional therapy: Including PT and OT, focused on the hands, wrist and forearm | 1hr, 5d/wk, for 4wk | Subacute  & Chronic subgroups |
| Chan et al.^58^  2018  China (Mainland) | Recovery in the severely impaired arm post-stroke after mirror therapy: a randomized controlled study | E: 15  C: 20 | Mirror therapy + Conventional Rehabilitation: Patients sat in front of a table on which a mirror was placed vertically in the space ipsilateral to the paretic arm. The mirror’s reflective surface was facing the intact arm. Patients were required to watch the reflective image of the whole intact arm from shoulder to the hand. Patients were instructed to practice 5 structured active movements with the intact arm. | Sham + Conventional Rehabilitation: Patients performed the same 5 structured exercises with both the paretic and intact arm but without a mirror. Conventional rehabilitation included PT and OT. The PT program included positioning, functional electrical stimulation, passive/assisted-active exercise on arm ergometers, and strengthening exercise. | 2x30min/d, 5d/wk for 4wk Mirror therapy  2.5h/d, 5d/wk Conventional rehabilitation | Acute |
| Kim et al.^59^  2016  South Korea | Effects of mirror therapy combined with motor tasks on upper extremity function and activities daily living of stroke patients | E: 12  C: 13 | Mirror therapy: Participants sat on chairs in front of a desk and bent their hip joints, knee joints, and ankle joints at 90°, with both feet on the floor at shoulder width. A mirror was stood on the desk, parallel with the median line of the body and facing the unaffected upper limb. | Conventional Therapy: Included training sessions for the improvement of upper limb function and activities of daily living. | 30min/d, 5dwk for 4wk | Chronic |
| Colomer et al.^60^  2016  Spain | Mirror therapy in chronic stroke survivors with severely impaired upper limb function: a randomized controlled trial | E: 15  C: 16 | Mirror therapy + Conventional therapy: A triangular prism-shaped device with a mirror on one side was fixed on a table, participants were encouraged to observe the mirror while executing movements. | Conventional Care: Included passive mobilization of the paretic upper limb | 45min/d, 3d/wk for 8wk | Chronic |
| Amasyali & Yaliman^61^  2016  Turkey | Comparison of the effects of mirror therapy and electromyography-triggered neuromuscular stimulation on hand functions in stroke patients: a pilot study. | E1: 9  C: 8  Multi-arm | Mirror therapy + Conventional Care: Patients practiced with the affected hand positioned behind the mirror while they looked at the reflection of the unaffected. Patients practiced their therapy at home after supervised sessions. | Conventional care: Consisted of conventional PT and OT, and included range of motion, stretching, and strengthening exercises. | 30min/d, 5d/wk for 3wk Mirror therapy  2hr/d Conventional care | Subacute |
| Lim et al.^62^  2016  South Korea | Efficacy of Mirror Therapy Containing Functional Tasks in Poststroke Patients | E: 30  C: 30 | Mirror Therapy: Patients underwent task-oriented mirror therapy by imitating the reflection of the normal upper limb in the mirror. | Sham: Patients performed the same functional tasks over the same period with a wood plate. Conventional therapy involved functional tasks with both hands without a mirror | 20min/d, 5d/wk for 4wk | Subacute |
| Gurbuz et al.^63^  2016  Turkey | Effect of mirror therapy on upper extremity motor function in stroke patients: A randomized controlled trial | E: 16  C: 15 | Mirror therapy + Conventional therapy: A mirror was placed between the extremities and vertical to the table in front of them so that the non-paretic hand was reflected. The patients were asked to perform periodic flexion and extension movements of the wrist and fingers on the non-paretic side and to observe the reflection of these movements in the mirror under supervision. | Sham+ Conventional therapy: During sham therapy the non-reflecting face of the mirror was used for an equal length of time, same exercises. Conventional therapy, consisted of neurodevelopmental facilitation techniques, such as range of motion exercises, strengthening exercises, and OT. | 20min/d, 5d/wk for 4 wk Mirror therapy  60-120min/d, 5d/wk for 4wk Conventional therapy | Subacute |
| Arya et al.^64^  2015  India | Task-Based Mirror Therapy Augmenting Motor Recovery in Poststroke Hemiparesis: A Randomized Controlled Trial | E: 17  C: 16 | Task-based Mirror Therapy + Conventional therapy: The less-affected upper limb was placed in front of the mirror. Tasks included elbow, forearm, wrist, and finger movements. | Conventional Therapy: The control group received conventional OT based on Brunnstrom and Bobath approaches. | 45min, 5d/wk, for 8wk mirror therapy  45min, 5d/wk, for 8wk conventional therapy | Chronic |
| Mirela Cristina et al.^65^  2015  Romania | Mirror therapy enhances upper extremity motor recovery in stroke patients. | E: 7  C: 8 | Mirror therapy + Conventional therapy: Patients observed the reflection in the mirror of their unaffected limb while performing movements with both arms. | Conventional therapy: Consisted of conventional stroke rehabilitation program for the upper limb, including neurorehabilitation techniques, electrical stimulation and OT | 30min/d, 5d/wk for 6wk mirror therapy  30min/d, 5d/wk for 6wk conventional therapy | Subacute |
| Samuelkamaleshkumar et al.^66^  2014  India | Mirror therapy enhances motor performance in the paretic upper limb after stroke: a pilot randomized controlled trial. | E: 10  C: 10 | Mirror Therapy + Conventional Care: Participants performed nonparetic wrist flexion, extension, radial and ulnar deviation, circumduction, fisting, releasing, abduction, and adduction of all fingers in front of the mirror box attempting the same movements with the paretic hand (bilateral arm training). | Conventional Rehabilitation: Participants of both the groups underwent a patient-specific multidisciplinary rehabilitation program involving conventional OT, PT, and speech therapy (if required). | 1hr, 5d/wk, for 3wk mirror therapy  6hr, 5d/wk, for 3wk Conventional rehabilitation | Acute |
| Wu et al.^67^  2013  Taiwan, China | Effects of mirror therapy on motor and sensory recovery in chronic stroke: a randomized controlled trial. | E: 16  C: 17 | Mirror therapy + Traditional activity: Participants were  instructed to look at the reflection of the unaffected hand in the mirror as if it were the affected hand and perform bilateral  symmetrical movements as much as possible. | Traditional therapeutic activities: Focused on improving motor control skills in the affected upper limb, coordination, and unilateral and bilateral fine motor tasks as well as enhancing static and dynamic standing and sitting, balance, and compensatory practice on functional tasks. | 60min/d, 5d/wk, 4wk  30min Traditional activity | Chronic |
| Thieme et al.^68^  2013  Germany | Mirror therapy for patients with severe arm paresis after stroke--a randomized controlled trial. | E1: 18  C: 21  Multi-arm | Individual Mirror Therapy: Patients were instructed to move both arms while looking in the mirror. The affected arm should be moved as well as possible. Isolated movements included: fingers, wrist, lower arm, elbow and shoulder joints. Object-related movements, included putting a ball or bigger squares in different directions, moving sticks or wipe-like movements with a cloth. | Sham: In this group the mirror was turned, so a wooden board restricted the view on the impaired arm. Patients in this group were instructed to move both arms while looking at the non-affected arm and imaging the analogous movements of the affected arm but then followed the same protocol as in the mirror therapy groups. | 30min/d, 20 sessions over 5wk | Subacute |
| Lee et al.^69^  2012  South Korea | The mirror therapy program enhances upper-limb motor recovery and motor function in acute stroke patients. | E: 13  C: 13 | Mirror therapy + Conventional Rehabilitation: Participants sat on a stool; a mirror was positioned perpendicular to the patient’s midline, and the affected hand was put  into a mirror box, whereas the unaffected hand was placed on the front of the reflective surface. | Conventional Rehabilitation: Standard rehabilitation comprised therapeutic exercise, OT, and functional electrical stimulation | 25min/d, 5d/wk for 4wk mirror therapy + Conventional Rehabilitation  75min/d, 5d/wk, 4wk Conventional Rehabilitation | Subacute |
| Michielsen et al.^70^  2011  Netherlands | Motor recovery and cortical reorganization after mirror therapy in chronic stroke patients: a phase II randomized controlled trial. | E: 17  C: 19 | Mirror therapy: Participants practiced with the affected hand positioned behind the mirror while they looked at the reflection of the unaffected hand in the mirror | Conventional therapy (sham exercise): Both groups performed bimanual exercises, with the difficulty of the exercises depending on the patients’ individual levels of functioning. The control group had a direct view of both hands | 1hr/d, 5d/wk (1d at rehabilitation centre, 5d at home), for 6wk | Chronic |
| **Motor Imagery** | | | | | | |
| Wang et al.^71^  2019  China (Mainland) | The Reorganization of Resting-State Brain Networks Associated With Motor Imagery Training in Chronic Stroke Patients. | E: 16  C: 15 | Motor Imagery + Conventional Rehabilitation: Patients performed specific motion imagery training of the impaired upper extremity. | Conventional Rehabilitation: Education on stroke information was provided, and conventional therapy included PT, OT, electrical stimulation, and Chinese acupuncture. | 30min/d, 5d/wk, for 4wk motor imagery  3h/d, 5d/wk, 4wk conventional rehabilitation | Subacute |
| Nam et al.^72^  2019  South Korea | Effects of adjuvant mental practice using inverse video of the unaffected upper limb in subacute stroke: a pilot randomized controlled study | E: 10  C: 10 | Mental practice + Conventional Rehabilitation: The training video consisted of six tasks derived from two assessment tools for poststroke patients: the manual function test and the Stroke Upper Limb Capacity Scale: grasp, carry a cube, pegboard, and pinch, sliding an object across a table and holding a cup. | Conventional Rehabilitation (30 min): rehabilitation therapy including proprioceptive exercises, verticalization, gait training, paretic hand and wrist mobilization, stretching and weight bearing of the affected arm, improving the range of motion of the affected arm, muscle strengthening, and the practice of tasks used for functional training. | 20min/d, 5d/wk, for 4wk mental practice  30min/d, 5d/wk, for 4wk Conventional rehabilitation | Subacute |
| Oh et al.^73^  2016  South Korea | Effects of Adjuvant Mental Practice on Affected Upper Limb Function Following a Stroke: Results of Three-Dimensional Motion Analysis, Fugl-Meyer Assessment of the Upper Extremity and Motor Activity Logs | E: 10  C: 10 | Mental Practice + Conventional rehabilitation: Patients were placed in a quiet, comfortable room and listened to a pre-recorded script directing them to imagine that they were moving the affected upper extremity. | Conventional rehabilitation: Components not specified | 20min, 3d/wk, 3wk mental imagery  30min, 5d/wk, 3wk Conventional rehabilitation | Subacute |
| Kim & Lee^74^  2015  South Korea | Motor imagery training improves upper extremity performance in stroke patients | E: 12  C: 12 | Motor Imagery+ Conventional PT: Patients sat comfortably in a chair and imagined a task related to daily living; the tasks involved imagining the sequence of movements that should be performed using their hands, such as drinking water from a cup, setting a seal, turning pages of a book, plugging a cord into an outlet, brushing their teeth, sorting chopsticks and spoons and putting them in a box, folding a towel, tearing off and folding a piece of toilet paper, making a phone call, placing a card in their wallet, etc. | Conventional PT: Components not specified | 30min/d, 5d/wk, for 4wk motor imagery  30min/d, 5d/wk, 4wk Conventional PT | Chronic |
| Park et al.^75^  2015  South Korea | Effects of mental practice on stroke patients' upper extremity function and daily activity performance. | E: 14  C: 15 | Mental practice + Conventional rehabilitation: The mental training comprised three stages: the preparation stage for mental practice; then, the training stage according to the actual task; and lastly, the organizing stage for mental practice training. | General rehabilitation: The control group received conventional OT and PT. | 10min/d, 5d/wk, for 2wk mental practice  30min/d, 5d/wk, for 2wk general rehabilitation | Chronic |
| Sun et al.^76^  2013  China (Mainland) | Cortical reorganization after motor imagery training in chronic stroke patients with severe motor impairment: a longitudinal fMRI study. | E: 9  C: 9 | Motor Imagery+ Conventional Rehabilitation Therapy: The program included relaxation in which patients were asked to imagine themselves in a warm, relaxing place (e.g., a beach) and asked to relax their muscles (i.e., progressive relaxation); patients imagined simple flexion and extension of the affected shoulder, elbow, wrist, and finger; as well as complex actions of activities of daily living. | Conventional Rehabilitation: Patients of both groups underwent standard rehabilitation that included PT and OT of the upper limbs (passive or active movement), electrical stimulation, Chinese acupuncture, and massage. | 30min/d, 5d/wk, 4wk motor imagery  3h/d, 5d/wk, 4wk conventional rehabilitation | Subacute |
| Page et al.^77^  2001  United States | A randomized efficacy and feasibility study of imagery in acute stroke. | E: 8  C: 5 | Mental Imagery + OT: Patients listened to a tape-recorded imagery intervention, which was read by a male psychologist and consisted of relaxation, asking patients to imagine themselves in a warm, relaxing place (e.g. a beach), and asking them to contract and relax their muscles (i.e. progressive relaxation). Suggestions for external, cognitive visual images related to using the affected arm in functional tasks were used. | OT: Exercises concentrated on the upper limbs, and for half an hour, exercises concentrated on the lower limbs, with some emphasis provided on gross arm movement. | 10min/d, 3d/wk, for 6wk mental imagery  1 hour, 3d/wk, for 6wk OT | Subacute |
| Page^78^  2000  United States | Imagery improves upper extremity motor function in chronic stroke patients: A pilot study | E: 8  C: 8 | Imagery training + OT: Patients were administered a tape-recorded imagery intervention consisting of relaxation followed by suggestions for external, cognitive visual images related to using the affected arm in weight-bearing tasks and functional tasks that were being practiced during their OT sessions. | OT: The therapists provided treatment that consisted of 40% neurodevelopmental techniques and 60% compensatory strategies using the unaffected limb. | 20min/d, 3d/wk, for 4wk imagery  30min/d, 3d/wk, for 4wk OT | Chronic |
| **Neuromuscular Electrical Stimulation (NMES)** | | | | | | |
| Zhou et al.^79^  2018  China (Mainland) | Efficiency of Neuromuscular Electrical Stimulation and Transcutaneous Nerve Stimulation on Hemiplegic Shoulder Pain: A Prospective Randomized Controlled Trial | E1: 31  C: 18  Multi-arm | NMES + Conventional Rehabilitation: NMES (15Hz and pulse width 200ms,  dual channel stimulators, rehabilitation kit) was applied to supraspinatus and deltoids (middle and posterior bundles). | Conventional Rehabilitation: The standardized program was delivered by occupational therapists and physical therapists. | 1hr, 5d/wk, for 4wk NMES  Timing and intensity of Conventional Rehabilitation not specified | Subacute |
| Amasyali & Yaliman^61^  2016  Turkey | Comparison of the effects of mirror therapy and electromyography-triggered neuromuscular stimulation on hand functions in stroke patients: a pilot study. | E2: 7  C: 8  Multi-arm | EMG-triggered NMES + Conventional Care: EMG-stimulation involved initiating a voluntary contraction for a specific movement until a threshold level is reached. Then, an additional electrical stimulus begins and the specific joint is subjected to a full range of motion. Patients were asked to perform maximum extension of their wrist several times to determine target stimulation. | Conventional Care: Patients received conventional PT during the same period. | 30min/d, 5d/wk for 3wk NMES  2hr/d approximately  Conventional care | Subacute |
| Kwakkel et al.^22^  2016  Netherlands | Effects of Unilateral Upper Limb Training in Two Distinct Prognostic Groups Early After Stroke: The EXPLICIT-Stroke Randomized Clinical Trial | E2: 50  C: 51  Multi-arm | EMG-NMES: Patients received stimulation of the finger extensors. Active participation of the patient was required to reach the EMG threshold during the dorsiflexion movement of fingers and wrist in order to trigger the NMS. EMG-NMS therapy was augmented by offering patients visual feedback of finger extension presented in front of the patient on a computer screen. | Usual care: Usual upper limb therapy in both strata consisted of exercise therapy based on recommendations from current Dutch guidelines. | 30min, 2x/d, 5d/wk, for 3wk EMG-NMES  30min, 5d/wk, for 3wk Usual care | Acute |
| Cui et al.^80^  2015  China (Mainland) | Effects of a 12-hour neuromuscular electrical stimulation treatment program on the recovery of upper extremity function in sub-acute stroke patients: a randomized controlled pilot trial | E2: 15  C: 15  Multi-arm | Short Duration NMES: A  portable surface neuromuscular stimulator (Chattanooga-2773AS) was used to deliver the NMES. Rectangular wave  pulsed currents (300μs pulse width; 40 Hz; 1 s on/off ramp)  were applied to the affected upper extremities. | Standard rehabilitation: Participants received rehabilitation that included PT, OT and activities of daily living training, mobility training, and speech therapy. | 30min, 6d/wk, for 4wk | Subacute |
| Lin & Yan^81^  2011  China (Mainland) | Long-term effectiveness of neuromuscular electrical stimulation for promoting motor recovery of the upper extremity after stroke. | E: 19  C: 18 | NMES + Standard Rehabilitation: The 2-channel Respond Select II  stimulator (Texas, USA) was used for NMES. The electrodes were applied over the motor points near the middle of the supraspinatus muscle and the deltoid muscle on the paretic side, as  well as over the wrist extensor. | Standard Rehabilitation: Included PT and OT | 30min/d, 5d/wk for 3wk NMES  30 min/d, 5d/wk for 3wk Standard rehabilitation | Subacute |
| Hsu et al.^82^  2010  Taiwan, China | Dose-response relation between neuromuscular electrical stimulation and upper-extremity function in patients with stroke. | E1: 22  E2: 22  C: 22  Multi-arm | High NMES + Conventional therapy: A portable surface neuromuscular stimulator with 2 channels (TENSMED-931) was used to deliver the NMES. The electrodes were placed over the extensor digitorum communis, extensor carpi radialis, flexor digitorum communis, supraspinatus, and posterior deltoid muscles.  E2: Low NMES + Conventional therapy: A portable surface neuromuscular stimulator with 2 channels (TENSMED-931) was used to deliver the NMES. The electrodes were placed over the extensor digitorum communis, extensor carpi radialis, flexor digitorum communis, supraspinatus, and posterior deltoid muscles. | Conventional therapy: All patients received regular inpatient rehabilitation | 60min, 5d/wk, for 4wks  Timing of conventional therapy not specified | Acute |
| Chae et al.^83^  1998  United Stares | Neuromuscular stimulation for upper extremity motor and functional recovery in acute hemiplegia | E: 14  C: 14 | NMES + Standard Rehabilitation: NMES was conducted with a surface neuromuscular stimulation unit (FOCUS, Empi Inc). Patients received stimulation of the extensor digitorum  communis and the extensor carpi radialis (ECR) through circular 2.5-cm surface electrodes | Sham NMES + Standard Rehabilitation: Participants received standard PT, OT, and speech therapy interventions as per routine of the inpatient stroke rehabilitation program. For sham, patients had the electrodes placed away from all motor points. | 1h/d, 15 sessions | Acute |
| **Rhythmic Auditory Stimulation (RAS)** | | | | | | |
| Tian et al.^84^  2020  China (Mainland) | Rhythmic Auditory Stimulation as an Adjuvant Therapy Improved Post-stroke Motor Functions of the Upper Extremity: A Randomized Controlled Pilot Study | E: 15  C: 15 | Rhythmic Auditory Stimulation: The RAS therapy was performed by practicing movements of certain tasks with auditory cues at a gradually increased rhythm. | Conventional Therapy: PT included strength, gait, balance, and coordination exercise. OT included forced use of the affected upper  extremity in activities of daily living, fine motor exercise of the hand, and sensory integration. | 30min/d, 5d/wk, for 4wk RAS  30min, 5d/wk for 4wk Conventional Therapy | Subacute |
| Chouhan & Kumar^85^  2012  India | Comparing the effects of rhythmic auditory cueing and visual cueing in acute hemiparetic stroke. | E1: 15  C: 15  Multi-arm | Rhythmic Auditory Stimulation + Conventional Treatment: Patients were instructed to do activities keeping pace with a metronome beat. | Conventional Treatment: including stretching of tightened muscles, gait training | 2h, 3x/wk for 3wk RAS  3wk Conventional Treatment | Subacute |
| **Robot-Assisted Training** | | | | | | |
| Lee et al.^86^  2021  Taiwan, China | Effects of Robot-Assisted Rehabilitation on Hand Function of People With Stroke: A Randomized, Crossover-Controlled, Assessor-Blinded Study. | E: 24  C: 24 | Robot-assisted Therapy (Gloreha Sinfonia Glove): Glove that detects individual finger movement and simulates activities of daily living function through task-oriented exercises. The robot focuses on the distal part of the upper limb and uses a dynamic support system to support the proximal part of the limb against gravity. | Conventional OT Program: Included task-oriented bilateral hand, grasp-and-release and pinch activities, after a 20-min warm-up program. | 60min, 2x/wk, for 6wk, 1mo washout | Chronic |
| Jiang et al.^87^  2021  China (Mainland) | Effects of short-term upper limb robot-assisted therapy on the rehabilitation of sub-acute stroke patients | E: 23  C: 22 | Robot-assisted Therapy (Armeo Spring) + Conventional Therapy: The robot provided shoulder, elbow, and wrist joint training using games in a projection display | Conventional Therapy: Including neurodevelopmental techniques, functional tasks, and muscle strengthening | 30min, 2x/d, 5d/wk for 2wk robot-assisted therapy  30min, 2x/d, 5d/wk for 2wk Conventional therapy | Acute |
| Ranzani et al.^88^  2020  Switzerland | Neurocognitive robot-assisted rehabilitation of hand function: a randomized control trial on motor recovery in subacute stroke. | E: 14  C: 13 | Robot-assisted Therapy (ReHapticKnob) + Conventional Neurocognitive Therapy: Haptic device used to train hand opening-closing and forearm pronation-supination integrating a set of seven exercises | Conventional Neurocognitive Therapy (dose-matched): Included sensorimotor and cognitive aspects, execution of complex tasks and activities of daily living | 3 sessions/d (2x45min, 1x30min) for 4wk | Acute |
| Xu et al.^89^  2020  China (Mainland) | Impact of smart force feedback rehabilitation robot training on upper limb motor function in the subacute stage of stroke | E: 20  C: 20 | Robot-assisted Therapy (Smart Force Feedback Rehabilitation Robot): Participants performed intensive functional training on the affected side through real-life mechanical scene simulations. | Conventional Therapy: Included traditional exercises such as stretching, roller training, and item transferring | 20mind/, 5d/wk, for 6wk | Subacute |
| Calabro et al.^90^  2019  Italy | Does hand robotic rehabilitation improve motor function by rebalancing interhemispheric connectivity after chronic stroke? Encouraging data from a randomised-clinical-trial. | E: 25  C: 25 | Robot-assisted Therapy (Amadeo Tyromotion Robot) + Conventional Rehabilitation: Robotic device that covers the hand fingers workspace and permits the movements of all fingers to provide repetitive and intensive training. | Conventional Rehabilitation (dose-matched: Included physiotherapy of the affected hand | 45min, 5d/wk for 8wk robot-assisted therapy  2.15h, 5d/wk for 8wk conventional rehabilitation | Chronic |
| Dehem et al.^91^  2019  Belgium | Effectiveness of upper-limb robotic-assisted therapy in the early rehabilitation phase after stroke: A single-blind, randomised, controlled trial | E: 15  C: 13 | Robot-assisted Therapy (REAplan robot) + Conventional care: Golf game involving the paretic hand along a trajectory while passing checkpoints. | Conventional Care: Included standard motor rehabilitation depending on patient needs and PT and OT as prescribed | 45min/d, 4d/wk, for 9wk (25% robot-assisted therapy and 75% conventional care) Robot group  45min/d, 4d/wk, for 9wk conventional care group | Acute |
| Rodgers et al.^92^  2019  United Kingdom | Robot assisted training for the upper limb after stroke (RATULS): a multicentre randomised controlled trial. | E: 232  C: 202 | Robot-assisted Therapy (MIT-Manus robotic gym system) + Usual Care: Robot-assisted arm movement integrating a shoulder-elbow module, wrist module, and hand module. | Usual care (as prescribed): Arm rehabilitation therapy log | 45min, 3x/wk, for 12wk | Chronic |
| Lee et al.^93^  2018  South Korea | Effects of robot-assisted therapy on upper extremity function and activities of daily living in hemiplegic patients: A single-blinded, randomized, controlled trial | E: 15  C: 15 | E: Robot-assisted Therapy (REJOYCE) + General OT: Robotic device for upper extremity comprising of a computer, screen and controller capable of moving in any direction in a three-dimensional space, and has nine types of manipulation functions necessary to perform activities of daily living | General OT: Included stretching, neurodevelopmental therapy, resistance exercise, and fine motor exercises such as cup stacking and ring insertion activities | 30min, 5x/wk for 8wk robot-assisted therapy  30min, 5x/wk for 8wk OT | Chronic |
| Daunoraviciene et al.^94^  2018  Lithuania | Effects of robot-assisted training on upper limb functional recovery during the rehabilitation of poststroke patients | E: 17  C: 17 | Robot-assisted Therapy (Armeo Spring): Robot that can provide shoulder, elbow, and wrist joint training, with five degrees of freedom that allows passive movement, games are displayed on a screen | Conventional Rehabilitation: Included exercising, physical activities, active table games | 30min, 5d/wk | Subacute |
| Tomic et al.^95^  2017  Serbia | ArmAssist robotic system versus matched conventional therapy for poststroke upper limb rehabilitation: A randomized clinical trial | E: 13  C: 13 | Robot-assisted Therapy (ArmAssist Robot): Robotic system developed for shoulder and elbow rehabilitation, combines a portable device for providing arm support over a table with interactive games with focus on reaching + Conventional rehabilitation | Conventional therapy: Included stretching, range of motion, active-assisted movements, functional tasks, activities of daily living, splinting/casting, endurance exercises, balance and gait training | 30min 5d/wk for 3wk robot-assisted therapy  30min, 5d/wk for 3wk conventional rehabilitation | Subacute |
| Fan et al.^96^  2016  Taiwan, China | Neural correlates of motor recovery after robot-assisted stroke rehabilitation: a case series study | E: 4  C: 2 | Robot-assisted arm Training (Bi-Manu-Track): Bilateral arm therapy in robotic arm system | Dose matched Training: Included functional task practice and motor facilitation, adapted according to the level of motor impairment and individual needs of the participants. | 90min/d, 5d/wk, for 4wk | Subacute |
| Susanto et al.^97^  2015  China (Mainland) | Efficacy of robot-assisted fingers training in chronic stroke survivors: a pilot randomized-controlled trial. | E: 9  C: 10 | Robot-assisted Therapy (hand exoskeleton): Provides assistance to the user's paretic hand to accomplish hand grasping and opening, and finger pinching | Sham: Non-assisted fingers training (Device disconnected) | 1hr/d, 5d/wk, for 4wk | Chronic |
| Prange et al.^98^  2015  Netherlands | The effect of arm support combined with rehabilitation games on upper-extremity function in subacute stroke: a randomized controlled trial. | E: 35  C: 33 | Robot-assisted Therapy with rehabilitation game (ArmeoBoom device): Arm support training setup with a sling suspension system and a screen that integrates a 3D virtual environment with computerized exercises/game | Conventional Therapy: Included standardized arm exercises, such as moving/stacking cups, placing disks, transporting blocks or pegs | 30min/d, 3x/wk, 6wk | Subacute |
| Masiero et al.^99^  2014  Italy | Randomized trial of a robotic assistive device for the upper extremity during early inpatient stroke rehabilitation. | E: 14  C: 16 | Robot-assisted Therapy (NeReBot): Robotic device for upper extremity based on direct drive wire actuation, it allows to train arm flexion/extension, pronation/supination, adduction/abduction, and circumduction. + Conventional Rehabilitation | Conventional Rehabilitation: Included Bobath technique exercises, proprioceptive exercises, functional re-education, gait training, occupational therapy and active/passive mobilization | 120min, 5d/wk for 5wk (65% conventional rehabilitation, 35% robot-assisted training)  120min, 5d/wk for 5wk Conventional rehabilitation | Acute |
| Klamroth-Marganska et al.^100^  2014  Switzerland | Three-dimensional, task-specific robot therapy of the arm after stroke: a multicentre, parallel-group randomised trial. | E: 38  C: 35 | Robot-assisted Therapy (Armin): Exoskeleton robot that allows task-specific training, therapy modes included mobilization, games and activities of daily living | Conventional Therapy: Involved PT and OT, and included mobilisation, games, activities of daily living, or any combination of the three | 45min, 3x/wk for 8wk | Chronic |
| Ang et al.^101^  2014  Singapore | Brain-computer interface-based robotic end effector system for wrist and hand rehabilitation: results of a three-armed randomized controlled trial for chronic stroke. | E2: 8  C: 7  Multi-arm | Robot-assisted Therapy (HapticKnob robotic arm): Robotic arm for hand grasping and knob manipulation for wrist pronation or supination | Standard arm therapy: including overall arm mobilization, forearm pronation-supination, wrist control and grasp-release of objects | 90min (60min robot-assisted therapy + 30min standard arm mobilization), 3x/wk for 6wk | Chronic |
| Brokaw et al.^102^  2014  United States | Robotic therapy provides a stimulus for upper limb motor recovery after stroke that is complementary to and distinct from conventional therapy. | E: 12  C: 10 | Robot-assisted therapy (ARMin and HandSOME devices): Combination of robotic devices to allow for simultaneous reach and grasp of virtual and real-world objects | Conventional Therapy: Included mobilization, stretching, range of motion | 12h therapy, 1mo washout | Chronic |
| Bartolo et al.^103^  2014  Italy | Arm weight support training improves functional motor outcome and movement smoothness after stroke. | E: 12  C: 16 | Robot-assisted Therapy (Armeo Spring): Robotic device with a spring mechanism allowing adjustable arm weight support | Conventional PT: Included passive mobilization, active motor exercises, proprioception, fine motor function of the hand | 90min/d, 6d/wk for 2wk (30min Robotic training + 60min PT  90min/d, 6d/wk for 2wk conventional PT | Acute |
| Yang et al.^104^  2012  Taiwan, China | Pilot comparative study of unilateral and bilateral robot-assisted training on upper-extremity performance in patients with stroke | E1: 7  E2:7  C: 7  Multi-arm | E1: Robot-assisted Therapy (Bi-Manu-Track) Unilateral training protocol: Robotic device that offers forearm pronation and supination and wrist flexion and extension, participants practiced with the paretic arm  E2: Robot-assisted Therapy (Bi-Manu-Track) Bilateral training protocol: Robotic device that offers forearm pronation and supination, wrist flexion and extension, participants practiced with both arms | Standard Rehabilitation: including weight bearing, stretching, strengthening of the paretic arm, coordination tasks, unilateral and bilateral fine motor tasks and balance | 90-105 min, 5d/wk for 4wk Robot Training  90-105min, 5d/wk for 4wk  Standard rehabilitation | Chronic |
| Reinkensmeyer et al.^105^  2012  United States | Comparison of three-dimensional, assist-as-needed robotic arm/hand movement training provided with Pneu-WREX to conventional tabletop therapy after chronic stroke. | E: 13  C: 13 | Robotic training (Pneu-WREX): Pneu-WREX is a 4 degree-of-freedom based on a passive arm support, pneumatically actuated upper limb orthosis for robot-aided movement training. Patients followed the movements on the computer screen. These movements involved reaching, grasping, grasp release, and horizontal movement. The robot provided assistance as needed for participants to complete the arm movements. | Conventional Exercise: Exercises were typical of conventional home programs and consisted of range of motion stretches, active range of motion strengthening exercises, and a list of activity of daily living tasks throughout the hemiparetic upper limb. | 1hr/d, 3d/wk for 8wk | Chronic |
| Conroy et al.^106^  2011  United States | Effect of gravity on robot-assisted motor training after chronic stroke: a randomized trial. | E1: 20  E2: 18  C: 19  Multi-arm | E1: Robot-assisted Therapy (InMotion 2.0 Shoulder/Arm Robot): Planar reaching focused on completion of shoulder and elbow movements toward visual targets.  E2: Robot-assisted Therapy (InMotion Linear Robot): Planar and vertical reaching against gravity | Conventional therapy: Including intensive active movement of the affected arm in a seated position | 1h, 3x/wk for 6wk | Chronic |
| Carmeli et al.^107^  2011  Israel | HandTutorTM enhanced hand rehabilitation after stroke—a pilot study | E: 16  C: 15 | Glove (HandTutor) + Traditional therapy: Impairment-oriented training system, exercises are based on repetitive and intensive active flexion and extension movements of the finger(s) and the wrist, and it is an ergonomic glove whose sensors allow the patient's movements to be monitored on a computer screen. | Traditional Therapy including active/passive exercises, range of motion, strength and endurance of wrist and fingers | 20-30min, for 5x/wk HandTutor  20-30min/d, for 3wk Traditional Therapy | Acute |
| Masiero & Armani^108^  2011  Italy | Upper-limb robot-assisted therapy in rehabilitation of acute stroke patients: focused review and results of new randomized controlled trial. | E: 11  C: 10 | Robotic-assisted Therapy (NeReBot): Robotic device for upper limb based on direct-drive wire actuation, and can help the patient perform spatial movements of shoulder and elbow. + Conventional Rehabilitation | Conventional Rehabilitation: Included proprioceptive exercises, functional re-education, gait training, occupational therapy, and passive/active mobilization | 120min, 5d/wk for 5wk (40min Robot therapy + 80min Conventional rehabilitation)  120min, 5d/wk for 5wk Conventional rehabilitation | Acute |
| Lo et al.^109^  2010  United States | Robot-assisted therapy for long-term upper-limb impairment after stroke. | E1: 25  C: 27  Multi-arm | Robot-assisted Therapy (MIT-Manus robotic system): Permits upper extremity flexion/extension, abduction/adduction, pronation/supination movements, and grasping exercises | Usual care: Included medical management, clinic visits as needed, and in some cases rehabilitation services | 36 sessions, 12wk | Chronic |
| Housman et al.^110^  2009  United States | A randomized controlled trial of gravity-supported, computer-enhanced arm exercise for individuals with severe hemiparesis. | E: 14  C: 14 | Robot-assisted Therapy (T-WREX): Arm orthosis robotic exoskeleton that provides weight support for the arm enabling naturalistic movement and can be used for computer game play, such as grocery shopping and cleaning games | Conventional Therapy: Included stretching, range of motion, strengthening exercises, activities of daily living with a tabletop for gravity support | 1hr, 3x/wk for 8-9wk | Chronic |
| Lum et al.^111^  2005  United States | The MIME robotic system for upper-limb neuro-rehabilitation: results from a clinical trial in subacute stroke | E1: 9  E2: 5  E3: 10  C: 6  Multi-arm | E1: Robot-assisted Therapy - Unilateral Robotic Training (MIME): Robotic arm training, only unilateral exercises.  E2: Robot-assisted Therapy - Bilateral Robotic Training (MIME): Robotic arm training, bilateral exercises.  E3: Robot-assisted Therapy - Combined Unilateral and Bilateral Robotic Training (MIME): Robotic arm training with both unilateral and bilateral exercises. | Conventional Therapy: Included proximal upper limb function exercises based on neurodevelopmental technique | 60min, 15 sessions for 4wk | Subacute |
| Lum et al.^112^  2002  United States | Robot-assisted movement training compared with conventional therapy techniques for the rehabilitation of upper-limb motor function after stroke. | E: 13  C: 14 | Robot (MIME) assisted movement training: Included passive mode, active-assisted mode, active-constrained mode, and bimanual mode. The robot assisted the affected limb by continuously moving the affected forearm to the contralateral forearm’s mirror-image position and orientation. | Conventional treatment + Sham: Targeted proximal upper-limb function that was based on neurodevelopmental therapy. Control participants received exposure to the robot for 5min within each session (sham). | 1hr/session, 24 session for 8wk | Chronic |
| **Sensory Stimulation** | | | | | | |
| Derakhshanfar et al.^113^  2021  Iran | Sensory interventions on motor function, activities of daily living, and spasticity of the upper limb in people with stroke: A randomized clinical trial | E: 30  C: 30 | Sensory Stimulation: Participants received randomly exteroceptive and proprioceptive stimulations. Stimulation included fast brushing, stretch pressure, and icing (Rood approach). | Conventional OT: Participants received the conventional OT including exercises for the improvement of upper extremity range of motion and fine movement and also strengthening upper extremity muscles | 45min/d, 4d/wk, for 6wk | Chronic |
| deDiego et al.^114^  2013  Spain | A sensorimotor stimulation program for rehabilitation of chronic stroke patients | E: 12  C: 9 | Sensory Stimulation: Included sensory stimulation training and functional activities; Patients had restricted use of the unaffected upper limb by using a rigid mitten that avoids both movement and sensory input to the hand. At home, patients participated in tactile stimulation with a toothbrush, mental imagination and activities of daily living, including grasping, reaching, handling, supporting, carrying. | Conventional Rehabilitation: The control group had the usual treatment according to the Bobath concept, without prioritizing therapy of the upper limb | 1hr/d, 2d/wk, 8wk | Chronic |
| **Task-Specific Training** | | | | | | |
| Arya et al.^115^  2012  India | Meaningful task-specific training (MTST) for stroke rehabilitation: a randomized controlled trial | E: 50  C: 52 | Task-specific training: Meaningful task-specific training, based on principles of motor learning, experience-dependent neuroplasticity, and shaping techniques. | Conventional Rehabilitation: Included Brunnstrom movement therapy and Bobath neurodevelopmental approach | 1h/d, 4-5d/wk for 4wk | Subacute |
| Winstein et al.^116^  2004  United States | A randomized controlled comparison of upper-extremity rehabilitation strategies in acute stroke: A pilot study of immediate and long-term outcomes | E2: 20  C: 20  Multi-arm | Functional Task Practice+ Standard Care: Task-specific functional training focused on the systematic and repetitive practice of tasks including pointing, grasping, stirring. | Standard care: Included muscle facilitation exercises emphasizing the neurodevelopmental approach, neuromuscular electric stimulation for shoulder subluxation, stretching exercises, and activities of daily living | 1d/h, 5d/wk for 4-6wk Functional task practice  4-6wk Standard care | Acute |
| Nelles et al.^117^  2001  Germany | Arm training induced brain plasticity in stroke studied with serial positron emission tomography. | E: 5  C: 5 | Task-oriented training: A special arm training for hemiparesis was adopted from task-oriented motor learning. Included repetitive passive elbow flexion and extension and intensive training with repetitive practice of active functional exercises that facilitate shoulder and proximal arm muscle activity. | Conventional Therapy: Included a nonspecific rehabilitation program including manual techniques such as range of motion exercises, soft tissue mobilization, and stretching exercises for the upper extremity musculature. | 45min/d, 4d/wk, for 3wk | Acute |
| **Theta Burst Stimulation (TBS)** | | | | | | |
| Khan et al.^118^  2019  India | The comparative efficacy of theta burst stimulation or functional electrical stimulation when combined with physical therapy after stroke: a randomized controlled trial. | E1: 20  C: 20  Multi-arm | Theta Burst Stimulation (TBS) + Standard PT: Surface electromyograms  were used to record from the first dorsal interossei  muscles bilaterally using Ag-AgCl electrodes with  a gain of 1 to 2 mv. Signals were filtered (bandwidth, 3Hz to 3 kHz) and then digitized by an analog-to-digital convertor with a 24-bit resolution  at a sampling rate of 5 kHz. | Standard PT: Included range of motion exercises, weight bearing, reaching, grasping, hold and release, activities of daily living | 3x/wk for 4 wk TBS  5x/wk for 1mo  Standard PT | Acute |
| Hsu et al.^119^  2013  Taiwan, China | Intermittent theta burst stimulation over ipsilesional primary motor cortex of subacute ischemic stroke patients: a pilot study. | E: 6  C: 6 | Intermittent Theta Burst Stimulation (iTBS) + Standard Rehabilitation: Consisted of a brief train of basic theta bursts (5 Hz) lasting for 2s (10 bursts, each burst containing three pulses of 50 Hz) given  every 10 s for 40 trains (a total of 1200 pulses, iTBS1200) at 80% the  intensity of the active motor thresholds. | Sham iTBS + Standard Rehabilitation: Included strength training for upper and lower extremities, and functional task training such as reaching, grasping, and hand ambulation | 10min, 1x/d, for 10d | Acute |
| **Transcutaneous Electrical Nerve Stimulation (TENS)** | | | | | | |
| Wu et al.^120^  2020  China (Mainland) | Effect and Safety of Transcutaneous Auricular Vagus Nerve Stimulation on Recovery of Upper Limb Motor Function in Subacute Ischemic Stroke Patients: A Randomized Pilot Study | E: 10  C: 11 | Transcutaneous Auricular Vagus Nerve Stimulation + Conventional Rehabilitation: Stimulation was applied by a BHD-1A transcutaneous electrical stimulation therapy instrument (Bohua, Weihai, Chi), pulse frequency was 20Hz. | Sham + Conventional Rehabilitation: Electrodes were fixed to the cymba conchae of the left ear without electrical stimulation. Conventional rehabilitation involved  postural control, proprioception, neuromuscular  facilitation, and gait training | 30 min/d, 15d stimulation  30 min/d, 15d Conventional rehabilitation | Subacute |
| Zhou et al.^79^  2018  China | Efficiency of Neuromuscular Electrical Stimulation and Transcutaneous Nerve Stimulation on Hemiplegic Shoulder Pain: A Prospective Randomized Controlled Trial | E2: 32  C: 18  Multi-arm | TENS + Conventional Rehabilitation: 100Hz and pulse width 100μs was applied to supraspinatus and deltoids (medial and posterior parts). | Conventional Rehabilitation: Standardized rehabilitation program delivered by occupational therapists and physical therapists. | 1hr, 5d/wk, for 4wk TENS  Duration of Conventional rehabilitation not specified | Subacute |
| **Virtual Reality (VR)** | | | | | | |
| Mekbib et al.^121^  2021  United States | A novel fully immersive virtual reality environment for upper extremity rehabilitation in patients with stroke | E: 12  C: 11 | Virtual Reality (MNVR-Rehab) + Occupational Therapy: Picking up, moving and placing virtual balls in a basket | Time-matched OT: Included activities of daily living, balance control, gait training, weight shift, and distal and proximal upper extremity functional movements | 1h/d, 4d/wk for 2wk | Subacute |
| Marques-Sule et al.^122^  2021  Spain | Effectiveness of Nintendo Wii and Physical Therapy in Functionality, Balance, and Daily Activities in Chronic Stroke Patients | E: 15  C: 14 | Virtual Reality (Nintendo Wii Balance Board) + Conventional PT: Wii Fit package, tennis, bowling, and golf games | Conventional PT: Including functional exercises, mobility and strength, balance, kinesiotherapy, coordination, walking exercises, weight transfer and training with obstacles | 30min 2x/wk for 4wk VR  35min, 2x/wk for 4wk Conventional PT | Chronic |
| Laffont et al.^123^  2020  France | Rehabilitation of the upper arm early after stroke: Video games versus conventional rehabilitation. A randomized controlled trial | E: 25  C: 26 | Non-immersive video gaming rehabilitation (VG) + Conventional rehabilitation: Slow and easy-to-play games with few distracters, requiring repetitive movements of the mouse and low cognitive demand | Conventional OT: Including intensive, repetitive and task-oriented movements | 15-45min, 5d/wk, for 6wk VG  90-180min/d, 5d/wk for 6wk Conventional OT | Acute |
| Kang et al.^124^  2020  South Korea | Effects of Upper-Extremity Rehabilitation Using Smart Glove in Patients With Subacute Stroke: Results of a Prematurely Terminated Multicenter Randomized Controlled Trial | E: 12  C: 11 | Smart Glove (RAPAEL) Training + Conventional Occupational Therapy: Monitors the movements of the fingers, hand and wrist, and motions included forearm supination/pronation, wrist flexion/extension, wrist radial/ulnar deviation and finger flexion/extension. Motion tasks were related to activities of daily living, such as painting fences. | Standard occupational therapy + upper-extremity self-training (homework): Tasks included grasping and releasing a grip ball, wiping a table using a soft towel, pushing a  rubber clay, putting  pins in diamond-shaped holes of a pegboard, etc. | 30min smart glove + 30min OT, 5d/wk for 2wk  30min upper extremity rehabilitation homework + 30min OT, 5d/wk for 2wk | Subacute |
| Long ^125^  2020  China (Mainland) | Effects of virtual reality training on occupational performance and self-efficacy of patients with stroke: a randomized controlled trial | E: 25  C: 27 | Virtual Reality (Doctor Kinetic) + Conventional Rehabilitation: Games require bilateral upper limb flexion and abduction activity | Conventional Rehabilitation: Including OT, PT, and acupuncture | 95min, 5x/wk, 3wk VR  45min/d, 5d/wk, 3wk Conventional rehabilitation | Acute |
| Norouzi-Gheidari et al.^126^  2020  Canada | Feasibility, safety and efficacy of a virtual reality EXERGAME system to supplement upper extremity rehabilitation post-stroke: a pilot randomized clinical trial and proof of principle. | E: 9  C: 9 | Virtual Reality (Jintronix) + Conventional Therapy: Tracing a horizontally or vertically oriented path, reaching for a target, moving hands together to catch, carry, and drop objects, clapping both hands to catch objects, and selecting and moving kitchen objects | Conventional Therapy: including OT and PT as prescribed | 30min, 2-3x/wk, for 4wk  4wk (as prescribed) Conventional Therapy | Chronic |
| Keskin et al.^127^  2020  Turkey | Efficacy of a video-based physical therapy and rehabilitation system in patients with post-stroke hemiplegia: A randomized, controlled, pilot study | E: 12  C: 12 | Virtual Reality (LeapMotion/Fizyosoft software) + Conventional Therapy: LeapBall and Pong games for upper extremity flexion-extension, supination-pronation | Conventional Therapy: Including PT and neurophysiological exercise program | 60min/d, 5d/wk, for 6wk | Chronic |
| Park et al.^128^  2019  South Korea | Effects of virtual reality-based planar motion exercises on upper extremity function, range of motion, and health-related quality of life: a multicenter, single-blinded, randomized, controlled pilot study. | E: 12  C: 13 | Virtual reality-based rehabilitation (Rapael Smart Board): Free exploration, Point-to-point reaching, circle drawing | Conventional OT: including range of motion exercises, figure tracing and cone stacking | 30min VR + 30min Conventional care, 5d/wk for 4wk  60min, 5d/wk for 4wk Conventional care only | Chronic |
| Oh et al.^129^  2019  South Korea | Efficacy of virtual reality combined with real instrument training for patients with stroke: a randomized controlled trial. | E: 17  C: 14 | Virtual Reality (Joystim): Instruments with 3 degrees of freedom, thumb pinch, doorknob, button, air tube, gas valve, tool turn, and steering wheel | Conventional therapy: Including standardized range of motion exercises, functional training, task-related exercises and activities of daily living, fine motor training, perception and cognition training | 30min/d, 3d/wk for 6wk | Chronic |
| Ogun et al.^130^  2019  Turkey | Effect of leap motion-based 3D immersive virtual reality usage on upper extremity function in ischemic stroke patients | E: 33  C: 32 | Virtual Reality (Leap Motion): Patients used the VR device to play task-oriented games that focused on gripping and handling of objects with arm and forearm motion and stability. Games included cube handling, decorating a tree with leaves and fruits, picking up vegetables from a bowl, kitchen experience game, drumming game, etc. | Sham: The control group received conventional upper extremity active exercises comprising the same tasks as used in the VR group. The control group also used the VR equipment, but only focused on visual scenes without any upper extremity interaction. | 15min, 3d/wk for 6wk VR/Sham  45min, 3d/wk for 6wk, Conventional therapy | Chronic |
| Henrique et al.^131^  2019  Brazil | Effects of Exergame on Patients' Balance and Upper Limb Motor Function after Stroke: A Randomized Controlled Trial | E: 16  C: 15 | Virtual Reality (Exergame Motion Rehab AVE 3D): Games for upper extremity and balance, including flexion exercises, shoulder abduction and adduction, elbow extension, wrist extension | Conventional PT: Composed of the same exercises employed in the experimental group, such as flexion exercises, shoulder abduction and adduction, abduction and horizontal shoulder adduction, elbow extension, wrist extension, knee flexion, hip flexion, and abduction | 30min, 2x/wk, for 12wk | Chronic |
| Hung et al.^132^  2019  Taiwan, China | Comparison of Kinect2Scratch game-based training and therapist-based training for the improvement of upper extremity functions of patients with chronic stroke: a randomized controlled single-blinded trial. | E: 17  C: 16 | Virtual Reality (Kinect system): Kinect2Scratch games group requiring the patient to move their affected limb, such as whack-a-mole, harvest carrots, picking apples, bowling etc. | Therapist-based training: Including bilateral or unilateral movement, single joint or multi-joint movements | 30min, 2-3x/wk, for 3mo | Chronic |
| Ikbali Afsar et al.^133^  2018  Turkey | Virtual reality in upper extremity rehabilitation of stroke patients: a randomized controlled trial | E: 19  C: 16 | Virtual Reality Training (Xbox Kinect) + Conventional Rehabilitation: Mouse mayhem game, traffic control, ballon buster, *mathercising* game from Dr. Kawashima’s Body and Brain Exercises package. | Conventional Rehabilitation: including position control, balance skills, weight shift, activities of daily living, proprioceptive neuromuscular facilitation and neurodevelopmental facilitation | 30min/d, 5x/wk, for 4wk VR  60min, 5x/wk, for 4wk Conventional Rehabilitation | Subacute |
| Faria et al.^134^  2018  Portugal | Combined Cognitive-Motor Rehabilitation in Virtual Reality Improves Motor Outcomes in Chronic Stroke - A Pilot Study | E: 12  C: 12 | Virtual reality (Reh@Task): Cancellation tests, including finding targets within a pool of distractors | Standard OT: including spatial and time orientation activities, and writing training | 45min, 3x/wk for 1mo | Chronic |
| Kim et al.^135^  2018  South Korea | A low cost Kinect-based virtual rehabilitation system for inpatient rehabilitation of the upper limb in patients with subacute stroke: A randomized, double-blind, sham-controlled pilot trial | E: 11  C: 8 | Virtual Reality (Kinect) + OT: Included “Push Museum,” “Apple Run,” and “Fruit Market” programs, made using the Unity three-dimensional (3D) game engine (Unity Technology  Inc., San Francisco, CA). | Sham + OT: Conventional rehabilitation included  gait training, swallowing training, and speech therapy during admission. Sham therapy was delivered using RehaCom (Hasomed  Inc., Magdeburg, Germany for cognitive training. | 30min/d, 5d/wk, for 10d VR/sham  30min/d, 5d/wk for 10d OT | Acute |
| Kiper et al.^136^  2018  Italy | Virtual Reality for Upper Limb Rehabilitation in Subacute and Chronic Stroke: A Randomized Controlled Trial. | E: 68  C: 68 | Reinforced Feedback in Virtual Environment (RFVE) + Conventional Rehabilitation: During the RFVE treatment, the patient was seated in front of a wall screen grasping a sensorized real object (e.g., ball, disk, glass) with the paretic hand, or using a fingerless glove. The object was matched to the virtual handling object. | Conventional Rehabilitation: Consisted  of exercises in many directions of the upper limb workspace (e.g., shoulder flexion  and extension, shoulder abduction and adduction, shoulder internal and external rotation, elbow flexion and extension, forearm  pronation and supination) and hand grasping-release tasks. | 1h/d, 5d/wk, for 4wk RFV  1h/d, 5d/wk, for 4wk Conventional Rehabilitation | Subacute |
| Askin et al.^137^  2018  Turkey | Effects of Kinect-based virtual reality game training on upper extremity motor recovery in chronic stroke | E: 18  C: 20 | Virtual Reality (Xbox Kinect): Good view hunting game, involving cleaning, and Hong Kong chef game, involving making food + Standardized Physical Therapy | Standard PT: Including range of motion exercises, strength, flexibility, transfer, posture, balance, coordination and activities of daily living | 1h/d, 5d/wk for 4wk VR  20 sessions, 5d/wk for 4wk Standard PT | Chronic |
| Kong et al.^138^  2016  Singapore | Efficacy of a virtual reality commercial gaming device in upper limb recovery after stroke: A randomized, controlled study | E: 33  C2: 35  Multi-arm | Virtual Reality Training (Nintendo Wii) + Conventional OT: Wii Sports and Sports Resort games, bowling, tennis, boxing, golf, baseball, table tennis, basketball, cycling, frisbee disk, swordplay, airplane flight control game | Conventional OT: Consisted of passive and active range of motion exercises, muscle strengthening, therapeutic stretching, and activities of daily living training. | 60min, 4x/wk for 3wk VR  60min, 5x/wk for 3wk Conventional OT | Acute |
| Lee et al.^139^  2016  South Korea | Canoe game-based virtual reality training to improve trunk postural stability, balance, and upper limb motor function in subacute stroke patients: a randomized controlled pilot study | E: 5  C: 5 | Virtual Reality (Nintendo Wii canoe game) + Conventional Rehabilitation: To create realistic effects (e.g., swaying from side to side), a canoe was made by attaching a chair to a springboard (width, 45 cm; diameter, 150cm; height, 20cm). | Conventional Rehabilitation: Including PT, OT, and functional electrical stimulation | 30min/d, 3x/wk, for 4wk VR  70min, 3x/wk, for 4wk conventional rehabilitation | Subacute |
| Seok et al.^140^  2016  South Korea | Can Short-Term Constraint-Induced Movement Therapy Combined With Visual Biofeedback Training Improve Hemiplegic Upper Limb Function of Subacute Stroke Patients? | E2: 10  C: 10  Multi-arm | Visual Biofeedback Training (VBT) (E-LINK system computer-generated games) + Conventional OT: Patients held a dynamometer and a pinchmeter and practiced flexing and extending their limbs while playing computer games. | Conventional OT: including fine motor exercises, strengthening exercises, range of motion exercises, and activities of daily living | 1h/d for 2wk VR  1hr/d for 2wk Conventional OT | Subacute |
| Shin et al.^141^  2016  South Korea | Effects of virtual reality-based rehabilitation on distal upper extremity function and health-related quality of life: A single-blinded, randomized controlled trial | E: 24  C: 22 | Smart Glove (RAPAEL) Training + Standard OT: Evaluates movements of the fingers, hand and wrist, and motions included forearm supination/pronation, wrist flexion/extension, wrist radial/ulnar deviation and finger flexion/extension. Games included simulation of activities of daily living such as squeezing oranges, pouring wine, painting fences, turning pages, cleaning the floor, etc. | Standard OT matched to VR exercises + Standard OT: Involved range of motion and strengthening exercises for the affected limb, tabletop activities, and training for activities of daily living | 30min/d, for 4wk VR  30min/d, for 4wk OT | Chronic |
| Da Silva Ribeiro et al.^142^  2015  Brazil | Virtual rehabilitation via Nintendo Wii® and conventional physical therapy effectively treat post-stroke hemiparetic patients | E: 15  C: 15 | Virtual Rehabilitation (Nintendo Wii): Tennis game, hula-hoop game, soccer and boxing games | Conventional PT: Including stretching, balance, mobilization, weight transfer, and gait training | 60min, 2x/wk for 2mo | Chronic |
| Shin et al.^143^  2015  South Korea | Effects of game-based virtual reality on health-related quality of life in chronic stroke patients: A randomized, controlled study. | E: 16  C: 16 | Virtual reality (RehabMaster): Games to encourage active arm and trunk movements + Conventional occupational therapy | C: Conventional OT: Including movement exercises such as reaching, catching, wrist flexion or extension, and grasping | 30min of VR + 30 min Conventional OT, 5d/wk for 4wk  1h, 5d/wk for 4wk  Conventional OT | Chronic |
| Shin et al.^144^  2014  South Korea | A task specific interactive game-based virtual reality rehabilitation system for patients with stroke: a usability test and two clinical experiments. | E: 9  C: 7 | Virtual reality (RehabMaster) Conventional OT: Underwater fire game, goalkeeper, bug hunter, rollercoaster game | Conventional OT: Used in the conventional clinical setting | 40min, 10 sessions for 2wk VR  20min, 10 sessions for 2wk conventional OT | Subacute |
| Choi et al.^145^  2014  South Korea | Effectiveness of commercial gaming-based virtual reality movement therapy on functional recovery of upper extremity in subacute stroke patients. | E: 10  C: 10 | Virtual Rehabilitation (Nintendo Wii): Swordplay, table tennis and canoe games. All the participants were instructed to play the games  with their hemiparetic upper extremity. | Conventional OT: Included goal-oriented and highly repetitive trainings. Therapy consisted of stretching and strengthening exercises using full range of motion of the upper extremity, fine motor training, and sensory motor recovery. | 30min/d, 5x/wk for 4wk | Acute |
| Yin et al.^146^  2014  Singapore | Virtual reality for upper extremity rehabilitation in early stroke: a pilot randomized controlled trial. | E: 11  C: 12 | Virtual Reality+ Conventional therapy: Virtual supermarket environment, picking fruit and placing in basket | Conventional Therapy: Including stretching, strength, balance, gait and functional training | 30min, 5d/wk, for 2wk | Acute |
| Kottink et al.^147^  2014  Netherlands | Gaming and Conventional Exercises for Improvement of Arm Function After Stroke: A Randomized Controlled Pilot Study | E: 8  C: 10 | Rehabilitation Videogame: *FurballHun*t involves chasing away birds by repeatedly reaching for them with the hand above the screen | Conventional Rehabilitation: Including reach exercises | 30min/d, 3d/wk, 6wk | Chronic |
| Kiper et al.^148^  2014  Italy | Reinforced feedback in virtual environment for rehabilitation of upper extremity dysfunction after stroke: preliminary data from a randomized controlled trial. | E: 23  C: 21 | Virtual Reality (VRRS system) + Traditional Rehabilitation: Moving an object following a virtual trajectory with obstacles. | Traditional Rehabilitation: including functional exercises, range of motion, grasping and strengthening exercises | 1h/d, 5d/wk for 4wk, VR  1h/d, 5d/wk for 4wk Traditional rehabilitation | Chronic |
| Friedman et al.^149^  2014  United States | Retraining and assessing hand movement after stroke using the MusicGlove: comparison with conventional hand therapy and isometric grip training. | E1: 12  C: 12 | MusicGlove: Instrumented glove that requires functional gripping movements to play music, colored notes scroll down in the computer on five distinct frets, hitting the note causes it to explore and increases the music volume. Correct notes are logged and displayed in a summary at the end of the game. | Conventional care: including range of motion, strengthening exercises, functional gripping practice and stretching | 1hr, 3x/wk, 2wk | Chronic |
| Sin & Lee^150^  2013  South Korea | Additional virtual reality training using Xbox Kinect in stroke survivors with hemiplegia. | E: 18  C: 17 | Virtual Reality Training (Xbox Kinect+ Conventional OT): Boxing and bowling in the Kinect sports pack, Rally Ball, 20,000 Leaks and Space Pop in the Kinect adventure pack, which all require the use of upper extremities | Conventional OT: including passive/active range of motion exercises, muscle strengthening, stretching, and activities of daily living training | 30min, 3x/wk for 6wk VR  30min, 3x/wk for 6wk OT | Chronic |
| Duff et al.^151^  2013  United States | Adaptive mixed reality rehabilitation improves quality of reaching movements more than traditional reaching therapy following stroke | E: 11  C: 10 | Adaptive mixed reality rehabilitation (real-time motion capture and smart objects): Reaching tasks with real-time feedback | Conventional PT: Including pegboard reaching tasks, bead threading, reaching tasks, range of motion and coordination exercises | 60min, 3x/wk for 4wk | Chronic |
| Kwon et al.^152^  2012  South Korea | Effects of virtual reality on upper extremity function and activities of daily living performance in acute stroke: a double-blind randomized clinical trial. | E: 13  C: 13 | Virtual Reality (IREX VR system) + Conventional Therapy: Bird and ball game, drum, coconutz game, soccer and conveyor games | Conventional therapy: including routine PT and OT, gait and balance training, table-top activities, strengthening exercises, and activities of daily living training | 30min/d, 5d/wk for 4wk VR  70min/d, 5d/wk for 4wk Conventional therapy | Acute |
| Levin et al.^153^  2012  Canada | Virtual reality versus conventional treatment of reaching ability in chronic stroke: clinical feasibility study. | E: 6  C: 6 | Virtual reality (Gesture Xtreme): Virtual supermarket, bird and balls game, soccer, volleyball, etc. | Conventional OT: Including reaching and holding objects | 45min/session, 9 sessions in total, 3wk | Chronic |
| In et al.^154^  2012  South Korea | Virtual reality reflection therapy improves motor recovery and motor function in the upper extremities of people with chronic stroke. | E: 11  C: 8 | Virtual Reality (Reflection therapy) + Conventional therapy: The affected hand was in a box while the other was placed directly under the camera, each participant had to line up his or her arm with the image of the other one displayed on the screen, the program included wrist flexion/extension, forearm pronation/supination, clenching and opening hand, picking cups, pegging clothespins, pushing buttons, using chopsticks, opening a bottle, puzzles, drawing and playing a game of toy golf | Sham + Conventional therapy: Sham involved the same treatment but patients had to look at their unaffected hand as the monitor was off. Conventional therapy components were not specified. | 30min/d, 5d/wk, 4wk | Chronic |
| Kiper et al.^155^  2011  Poland | The effectiveness of reinforced feedback in virtual environment in the first 12 months after stroke. | E: 40  C: 40 | Reinforced Feedback in Virtual Environment (RFVE) + Traditional Neuromotor Rehabilitation: During the virtual therapy the participant was seated in front of the wall screen grasping a sensorized real object (ball, disc or cube) with the affected hand. If the grasp was not possible the sensors were fixed on a glove worn by the patient. Tasks consisted of simple movements, e.g. pouring water from a glass, using a hammer, turning around the centre of a doughnut, etc. | Traditional Neuromotor Rehabilitation: Including exercises based on traditional rehabilitation after stroke, postural control, functional skills, proximal-distal exercises, coordination, and grasping-release exercises | 1h RFVE, 5d/wk, for 4wk  1h, 5d/wk for 4wk Traditional Rehabilitation | Subacute |
| Piron et al.^156^  2010  Italy | Motor learning principles for rehabilitation: a pilot randomized controlled study in poststroke patients. | E: 27  C: 23 | Reinforced Feedback in Virtual Environment (RFVE): Manipulating objects, following trajectories displayed on the screen. | Conventional Therapy: Including exercises based on Bobath principles. | 1h/d, 5d/wk for 4wk | Chronic |
| Piron et al.^157^  2009  Italy | Exercises for paretic upper limb after stroke: a combined virtual-reality and telemedicine approach. | E: 18  C: 18 | Home-Based Virtual Reality (VRRS system): Moving an object following a trajectory displayed on the computer screen. | Traditional PT: Patients were asked to perform specific exercises for the upper limb with a strategy of progressive complexity. They were requested to control isolated motions without postural control, then postural control was included and, finally, complex motion with postural control was practiced. | 60min/d, 5d/wk, for 1mo | Chronic |

**Abbreviations:** AO=Action Observation; BAT=Bilateral Arm Training; tDCS=Transcranial Direct Current Stimulation; rTMS=Repetitive Transcranial Magnetic Stimulation; PT=Physiotherapy/Physical Therapy; OT=Occupational Therapy; CIMT=Constraint-Induced Therapy; mCIMT=Modified Constraint-Induced Therapy; EEG=Electroencephalogram; cm=centimetre(s); mA=milliamperes; x=times; wks=week(s); d=day(s); hr=hour(s); min=minute(s); Hz=Hertz; MEPs=Motor-Evoked Potentials; mm=milometers; NMES=Neuromuscular Electrical Stimulation; EMG=Electromyography; μs=Microsecond; RAS=Rhythmic Auditory Stimulation; TBS=Theta Burst Stimulation; iTBS=Intermittent Theta Burst Stimulation; TENS=Transcutaneous Electrical Nerve Stimulation; VR=Virtual Reality; VG=Video game(s); RFVE=Reinforced Feedback in Virtual Environment.

**List of Included Randomized Controlled Trials**

1. Zhu MH, Zeng M, Shi MF, et al. Visual feedback therapy for restoration of upper limb function of stroke patients. *Int J Nurs Sci* 2020; 7: 170-178. 2020/07/21. DOI: 10.1016/j.ijnss.2020.04.004.

2. Fu J, Zeng M, Shen F, et al. Effects of action observation therapy on upper extremity function, daily activities and motion evoked potential in cerebral infarction patients. *Medicine* 2017; 96: e8080.

3. Zhu M-H, Wang J, Gu X-D, et al. Effect of action observation therapy on daily activities and motor recovery in stroke patients. *Int J Nurs Sci* 2015; 2: 279-282.

4. Kim JH, Han JY, Song MK, et al. Synergistic Effects of Scalp Acupuncture and Repetitive Transcranial Magnetic Stimulation on Cerebral Infarction: A Randomized Controlled Pilot Trial. *Brain Sci* 2020; 10 2020/02/13. DOI: 10.3390/brainsci10020087.

5. Wang H-Q, Hou M, Li H, et al. Effects of acupuncture treatment on motor function in patients with subacute hemorrhagic stroke: a randomized controlled study. *Complement Ther Med* 2020; 49: 102296.

6. Zhang Y, Al-Aref R, Fu H, et al. Neuronavigation-Assisted Aspiration and Electro-Acupuncture for Hypertensive Putaminal Hemorrhage: A Suitable Technique on Hemiplegia Rehabilitation. *Turk Neurosurg* 2017; 27: 500-508. 2016/09/07. DOI: 10.5137/1019-5149.Jtn.16456-15.1.

7. Hsieh RL, Wang LY and Lee WC. Additional therapeutic effects of electroacupuncture in conjunction with conventional rehabilitation for patients with first-ever ischaemic stroke. *J Rehabil Med* 2007; 39: 205-211. 2007/05/01. DOI: 10.2340/16501977-0032.

8. Alexander DN, Cen S, Sullivan KJ, et al. Effects of acupuncture treatment on poststroke motor recovery and physical function: a pilot study. *Neurorehabil Neural Repair* 2004; 18: 259-267.

9. Lee S, Song B and Kim H. The effect of bilateral upper limb training on the recovery of upper limb function in patients with acute stroke. *Med Leg Update* 2019; 19: 515-520.

10. Lee MJ, Lee JH, Koo HM, et al. Effectiveness of Bilateral Arm Training for Improving Extremity Function and Activities of Daily Living Performance in Hemiplegic Patients. *J Stroke Cerebrovasc Dis* 2017; 26: 1020-1025. 2017/02/07. DOI: 10.1016/j.jstrokecerebrovasdis.2016.12.008.

11. Lin CH, Chou LW, Luo HJ, et al. Effects of Computer-Aided Interlimb Force Coupling Training on Paretic Hand and Arm Motor Control following Chronic Stroke: A Randomized Controlled Trial. *PLoS One* 2015; 10: e0131048. 2015/07/21. DOI: 10.1371/journal.pone.0131048.

12. Lin KC, Chen YA, Chen CL, et al. The effects of bilateral arm training on motor control and functional performance in chronic stroke: a randomized controlled study. *Neurorehabil Neural Repair* 2010; 24: 42-51. 2009/09/05. DOI: 10.1177/1545968309345268.

13. Lin KC, Chang YF, Wu CY, et al. Effects of constraint-induced therapy versus bilateral arm training on motor performance, daily functions, and quality of life in stroke survivors. *Neurorehabil Neural Repair* 2009; 23: 441-448. 2009/01/02. DOI: 10.1177/1545968308328719.

14. Desrosiers J, Bourbonnais D, Corriveau H, et al. Effectiveness of unilateral and symmetrical bilateral task training for arm during the subacute phase after stroke: a randomized controlled trial. *Clin Rehabil* 2005; 19: 581-593. 2005/09/27. DOI: 10.1191/0269215505cr896oa.

15. Alisar DC, Ozen S and Sozay S. Effects of Bihemispheric Transcranial Direct Current Stimulation on Upper Extremity Function in Stroke Patients: A randomized Double-Blind Sham-Controlled Study. *J Stroke Cerebrovasc Dis* 2020; 29: 104454. 2019/11/09. DOI: 10.1016/j.jstrokecerebrovasdis.2019.104454.

16. Lee D-G and Lee D-Y. Effects of adjustment of transcranial direct current stimulation on motor function of the upper extremity in stroke patients. *J Phys Ther Sci* 2015; 27: 3511-3513.

17. Fusco A, Assenza F, Iosa M, et al. The ineffective role of cathodal tDCS in enhancing the functional motor outcomes in early phase of stroke rehabilitation: an experimental trial. *Biomed Res Int* 2014; 2014: 547290. 2014/06/05. DOI: 10.1155/2014/547290.

18. Wu D, Qian L, Zorowitz RD, et al. Effects on decreasing upper-limb poststroke muscle tone using transcranial direct current stimulation: a randomized sham-controlled study. *Arch Phys Med Rehabil* 2013; 94: 1-8. 2012/08/11. DOI: 10.1016/j.apmr.2012.07.022.

19. Nair DG, Renga V, Lindenberg R, et al. Optimizing recovery potential through simultaneous occupational therapy and non-invasive brain-stimulation using tDCS. *Restor Neurol Neurosci* 2011; 29: 411-420. 2011/11/30. DOI: 10.3233/rnn-2011-0612.

20. Abdullahi A. Effects of Number of Repetitions and Number of Hours of Shaping Practice during Constraint‐Induced Movement Therapy: A Randomized Controlled Trial. *Neurol Res Int* 2018; 2018: 5496408.

21. Yadav RK, Sharma R, Borah D, et al. Efficacy of modified constraint induced movement therapy in the treatment of hemiparetic upper limb in stroke patients: a randomized controlled trial. *J Clin Med* 2016; 10: YC01.

22. Kwakkel G, Winters C, Van Wegen EE, et al. Effects of unilateral upper limb training in two distinct prognostic groups early after stroke: the EXPLICIT-stroke randomized clinical trial. *Neurorehabil Neural Repair* 2016; 30: 804-816.

23. Thrane G, Askim T, Stock R, et al. Efficacy of Constraint-Induced Movement Therapy in Early Stroke Rehabilitation: A Randomized Controlled Multisite Trial. *Neurorehabil Neural Repair* 2015; 29: 517-525. 2014/11/16. DOI: 10.1177/1545968314558599.

24. El-Helow MR, Zamzam ML, Fathalla MM, et al. Efficacy of modified constraint-induced movement therapy in acute stroke. *Eur J Phys Rehabil Med* 2015; 51: 371-379. 2014/07/18.

25. Yoon JA, Koo BI, Shin MJ, et al. Effect of constraint-induced movement therapy and mirror therapy for patients with subacute stroke. *Ann Rehabil Med* 2014; 38: 458-466. 2014/09/18. DOI: 10.5535/arm.2014.38.4.458.

26. van Delden AL, Peper CL, Nienhuys KN, et al. Unilateral versus bilateral upper limb training after stroke: the Upper Limb Training After Stroke clinical trial. *Stroke* 2013; 44: 2613-2616. 2013/07/23. DOI: 10.1161/strokeaha.113.001969.

27. Singh P and Pradhan B. Study to assess the effectiveness of modified constraint-induced movement therapy in stroke subjects: A randomized controlled trial. *Ann Indian Acad Neurol* 2013; 16: 180-184. 2013/08/21. DOI: 10.4103/0972-2327.112461.

28. Wu CY, Chen YA, Chen HC, et al. Pilot trial of distributed constraint-induced therapy with trunk restraint to improve poststroke reach to grasp and trunk kinematics. *Neurorehabil Neural Repair* 2012; 26: 247-255. 2011/09/10. DOI: 10.1177/1545968311415862.

29. Lin KC, Chung HY, Wu CY, et al. Constraint-induced therapy versus control intervention in patients with stroke: a functional magnetic resonance imaging study. *Am J Phys Med Rehabil* 2010; 89: 177-185. 2010/02/23. DOI: 10.1097/PHM.0b013e3181cf1c78.

30. Lin KC, Wu CY, Liu JS, et al. Constraint-induced therapy versus dose-matched control intervention to improve motor ability, basic/extended daily functions, and quality of life in stroke. *Neurorehabil Neural Repair* 2009; 23: 160-165. 2008/11/05. DOI: 10.1177/1545968308320642.

31. Page SJ, Levine P, Leonard A, et al. Modified constraint-induced therapy in chronic stroke: results of a single-blinded randomized controlled trial. *Phys Ther* 2008; 88: 333-340. 2008/01/05. DOI: 10.2522/ptj.20060029.

32. Lin KC, Wu CY and Liu JS. A randomized controlled trial of constraint-induced movement therapy after stroke. *Acta Neurochir Suppl* 2008; 101: 61-64. 2008/07/23. DOI: 10.1007/978-3-211-78205-7_10.

33. Boake C, Noser EA, Ro T, et al. Constraint-induced movement therapy during early stroke rehabilitation. *Neurorehabil Neural Repair* 2007; 21: 14-24. 2006/12/19. DOI: 10.1177/1545968306291858.

34. Wu CY, Chen CL, Tang SF, et al. Kinematic and clinical analyses of upper-extremity movements after constraint-induced movement therapy in patients with stroke: a randomized controlled trial. *Arch Phys Med Rehabil* 2007; 88: 964-970. 2007/08/07. DOI: 10.1016/j.apmr.2007.05.012.

35. Wu CY, Chen CL, Tsai WC, et al. A randomized controlled trial of modified constraint-induced movement therapy for elderly stroke survivors: changes in motor impairment, daily functioning, and quality of life. *Arch Phys Med Rehabil* 2007; 88: 273-278. 2007/02/27. DOI: 10.1016/j.apmr.2006.11.021.

36. Page SJ, Levine P and Leonard AC. Modified constraint-induced therapy in acute stroke: a randomized controlled pilot study. *Neurorehabil Neural Repair* 2005; 19: 27-32. 2005/01/28. DOI: 10.1177/1545968304272701.

37. Mahdy Ibrahim E, Ahmed Zaki M and Gaber Mahmoud Gabr M. Effect of High Frequency Repetitive Transcranial Magnetic Stimulation of the Contralesional Motor Cortex on Recovery from Post-Stroke Sever Motor Impairment. *AMJ* 2020; 49: 651-666. DOI: 10.21608/amj.2020.70959.

38. Guan YZ, Li J, Zhang XW, et al. Effectiveness of repetitive transcranial magnetic stimulation (rTMS) after acute stroke: A one-year longitudinal randomized trial. *CNS Neurosci Ther* 2017; 23: 940-946. 2017/10/04. DOI: 10.1111/cns.12762.

39. Li J, Meng XM, Li RY, et al. Effects of different frequencies of repetitive transcranial magnetic stimulation on the recovery of upper limb motor dysfunction in patients with subacute cerebral infarction. *Neural Regen Res* 2016; 11: 1584-1590. 2016/12/03. DOI: 10.4103/1673-5374.193236.

40. Sharma H, Vishnu V, Kumar N, et al. Efficacy of low-frequency repetitive transcranial magnetic stimulation in ischemic stroke: a double-blind randomized controlled trial. *Arch Phys Med Rehabil* 2020; 2: 100039.

41. El-Tamawy MS, Darwish MH, Elkholy SH, et al. Effect of repetitive transcranial magnetic stimulation on cortical and motor outcomes post stroke: a randomized controlled trial. *Indian J Public Health* 2019; 10: 215.

42. Long H, Wang H, Zhao C, et al. Effects of combining high- and low-frequency repetitive transcranial magnetic stimulation on upper limb hemiparesis in the early phase of stroke. *Restor Neurol Neurosci* 2018; 36: 21-30. 2018/02/15. DOI: 10.3233/rnn-170733.

43. Harvey RL, Edwards D, Dunning K, et al. Randomized Sham-Controlled Trial of Navigated Repetitive Transcranial Magnetic Stimulation for Motor Recovery in Stroke. *Stroke* 2018; 49: 2138-2146. 2018/10/26. DOI: 10.1161/strokeaha.117.020607.

44. Yang NY, Fong KN, Li-Tsang CW, et al. Effects of repetitive transcranial magnetic stimulation combined with sensory cueing on unilateral neglect in subacute patients with right hemispheric stroke: a randomized controlled study. *Clin Rehabil* 2017; 31: 1154-1163. 2016/12/07. DOI: 10.1177/0269215516679712.

45. Tosun A, Türe S, Askin A, et al. Effects of low-frequency repetitive transcranial magnetic stimulation and neuromuscular electrical stimulation on upper extremity motor recovery in the early period after stroke: a preliminary study. *Top Stroke Rehabil* 2017; 24: 361-367. 2017/03/23. DOI: 10.1080/10749357.2017.1305644.

46. Aşkın A, Tosun A and Demirdal Ü S. Effects of low-frequency repetitive transcranial magnetic stimulation on upper extremity motor recovery and functional outcomes in chronic stroke patients: A randomized controlled trial. *Somatosens Mot Res* 2017; 34: 102-107. 2017/04/22. DOI: 10.1080/08990220.2017.1316254.

47. Hosomi K, Morris S, Sakamoto T, et al. Daily repetitive transcranial magnetic stimulation for poststroke upper limb paresis in the subacute period. *J Stroke Cerebrovasc Dis* 2016; 25: 1655-1664.

48. Barros Galvão SC, Borba Costa dos Santos R, Borba dos Santos P, et al. Efficacy of coupling repetitive transcranial magnetic stimulation and physical therapy to reduce upper-limb spasticity in patients with stroke: a randomized controlled trial. *Arch Phys Med Rehabil* 2014; 95: 222-229. 2013/11/19. DOI: 10.1016/j.apmr.2013.10.023.

49. Etoh S, Noma T, Ikeda K, et al. Effects of repetitive trascranial magnetic stimulation on repetitive facilitation exercises of the hemiplegic hand in chronic stroke patients. *J Rehabil Med* 2013; 45: 843-847. 2013/07/03. DOI: 10.2340/16501977-1175.

50. Seniów J, Bilik M, Leśniak M, et al. Transcranial magnetic stimulation combined with physiotherapy in rehabilitation of poststroke hemiparesis: a randomized, double-blind, placebo-controlled study. *Neurorehabil Neural Repair* 2012; 26: 1072-1079. 2012/05/17. DOI: 10.1177/1545968312445635.

51. Chinnavan E, Ragupathy R and Wah YC. Effectiveness of mirror therapy on upper limb motor functions among hemiplegic patients. *Bangladesh J Med Sci* 2020; 19: 208-213.

52. Madhoun HY, Tan B, Feng Y, et al. Task-based mirror therapy enhances the upper limb motor function in subacute stroke patients: a randomized control trial. *Eur J Phys Rehabil Med* 2020; 56: 265-271.

53. Guo J, Qian S, Wang Y, et al. Clinical study of combined mirror and extracorporeal shock wave therapy on upper limb spasticity in poststroke patients. *Int J Rehabil Res* 2019; 42: 31-35.

54. Bai Z, Zhang J, Zhang Z, et al. Comparison between movement-based and task-based mirror therapies on improving upper limb functions in patients with stroke: a pilot randomized controlled trial. *Front Neurol* 2019; 10: 288.

55. Antoniotti P, Veronelli L, Caronni A, et al. No evidence of effectiveness of mirror therapy early after stroke: an assessor-blinded randomized controlled trial. *Clin Rehabil* 2019; 33: 885-893.

56. Ding L, Wang X, Chen S, et al. Camera-based mirror visual input for priming promotes motor recovery, daily function, and brain network segregation in subacute stroke patients. *Neurorehabil Neural Repair* 2019; 33: 307-318.

57. Ding L, Wang X, Guo X, et al. Camera-based mirror visual feedback: potential to improve motor preparation in stroke patients. *IEEE Trans Neural Syst Rehabil Eng* 2018; 26: 1897-1905.

58. Chan WC and Au-Yeung SS. Recovery in the severely impaired arm post-stroke after mirror therapy: a randomized controlled study. *Am J Phys Med Rehabil* 2018; 97: 572-577.

59. Kim K, Lee S, Kim D, et al. Effects of mirror therapy combined with motor tasks on upper extremity function and activities daily living of stroke patients. *J Phys Ther Sci* 2016; 28: 483-487. 2016/04/12. DOI: 10.1589/jpts.28.483.

60. Colomer C, Noe E and Llorens Rodríguez R. Mirror therapy in chronic stroke survivors with severely impaired upper limb function: a randomized controlled trial. *Eur J Phys Rehabil Med* 2016; 52: 271-278.

61. Amasyali SY and Yaliman A. Comparison of the effects of mirror therapy and electromyography-triggered neuromuscular stimulation on hand functions in stroke patients: a pilot study. *Int J Rehabil Res* 2016; 39: 302-307.

62. Lim K-B, Lee H-J, Yoo J, et al. Efficacy of mirror therapy containing functional tasks in poststroke patients. *Ann Rehabil Med* 2016; 40: 629-636.

63. Gurbuz N, Afsar SI, Ayaş S, et al. Effect of mirror therapy on upper extremity motor function in stroke patients: a randomized controlled trial. *J Phys Ther Sci* 2016; 28: 2501-2506.

64. Arya KN, Pandian S, Kumar D, et al. Task-based mirror therapy augmenting motor recovery in poststroke hemiparesis: a randomized controlled trial. *J Stroke Cerebrovasc Dis* 2015; 24: 1738-1748.

65. Mirela Cristina L, Matei D, Ignat B, et al. Mirror therapy enhances upper extremity motor recovery in stroke patients. *Acta Neurol Belg* 2015; 115: 597-603.

66. Samuelkamaleshkumar S, Reethajanetsureka S, Pauljebaraj P, et al. Mirror therapy enhances motor performance in the paretic upper limb after stroke: a pilot randomized controlled trial. *Arch Phys Med Rehabil* 2014; 95: 2000-2005.

67. Wu CY, Huang PC, Chen YT, et al. Effects of mirror therapy on motor and sensory recovery in chronic stroke: a randomized controlled trial. *Arch Phys Med Rehabil* 2013; 94: 1023-1030. 2013/02/20. DOI: 10.1016/j.apmr.2013.02.007.

68. Thieme H, Bayn M, Wurg M, et al. Mirror therapy for patients with severe arm paresis after stroke–a randomized controlled trial. *Clin Rehabil* 2013; 27: 314-324.

69. Lee MM, Cho H-y and Song CH. The mirror therapy program enhances upper-limb motor recovery and motor function in acute stroke patients. *Am J Phys Med Rehabil* 2012; 91: 689-700.

70. Michielsen ME, Selles RW, Van Der Geest JN, et al. Motor recovery and cortical reorganization after mirror therapy in chronic stroke patients: a phase II randomized controlled trial. *Neurorehabil Neural Repair* 2011; 25: 223-233.

71. Wang H, Xu G, Wang X, et al. The Reorganization of Resting-State Brain Networks Associated With Motor Imagery Training in Chronic Stroke Patients. *IEEE Trans Neural Syst Rehabil Eng* 2019; 27: 2237-2245. 2019/09/20. DOI: 10.1109/tnsre.2019.2940980.

72. Nam JS, Im Yi T and Im Moon H. Effects of adjuvant mental practice using inverse video of the unaffected upper limb in subacute stroke: a pilot randomized controlled study. *Int J Rehabil Res* 2019; 42: 337-343.

73. Oh HS, Kim EJ, Kim DY, et al. Effects of adjuvant mental practice on affected upper limb function following a stroke: results of three-dimensional motion analysis, fugl-meyer assessment of the upper extremity and motor activity logs. *Annals of rehabilitation medicine* 2016; 40: 401-411.

74. Kim S-S and Lee B-H. Motor imagery training improves upper extremity performance in stroke patients. *J Phys Ther Sci* 2015; 27: 2289-2291.

75. Park J, Lee N, Cho M, et al. Effects of mental practice on stroke patients' upper extremity function and daily activity performance. *J Phys Ther Sci* 2015; 27: 1075-1077. 2015/05/23. DOI: 10.1589/jpts.27.1075.

76. Sun L, Yin D, Zhu Y, et al. Cortical reorganization after motor imagery training in chronic stroke patients with severe motor impairment: a longitudinal fMRI study. *Neuroradiology* 2013; 55: 913-925.

77. Page SJ, Levine P, Sisto S, et al. A randomized efficacy and feasibility study of imagery in acute stroke. *Clinical rehabilitation* 2001; 15: 233-240.

78. Page SJ. Imagery improves upper extremity motor function in chronic stroke patients: a pilot study. *Occup Ther J Res* 2000; 20: 200-215.

79. Zhou M, Li F, Lu W, et al. Efficiency of Neuromuscular Electrical Stimulation and Transcutaneous Nerve Stimulation on Hemiplegic Shoulder Pain: A Randomized Controlled Trial. *Arch Phys Med Rehabil* 2018; 99: 1730-1739. 2018/05/20. DOI: 10.1016/j.apmr.2018.04.020.

80. Cui BJ, Wang DQ, Qiu JQ, et al. Effects of a 12-hour neuromuscular electrical stimulation treatment program on the recovery of upper extremity function in sub-acute stroke patients: a randomized controlled pilot trial. *J Phys Ther Sci* 2015; 27: 2327-2331. 2015/08/28. DOI: 10.1589/jpts.27.2327.

81. Lin Z and Yan T. Long-term effectiveness of neuromuscular electrical stimulation for promoting motor recovery of the upper extremity after stroke. *J Rehabil Med* 2011; 43: 506-510. 2011/05/03. DOI: 10.2340/16501977-0807.

82. Hsu SS, Hu MH, Wang YH, et al. Dose-response relation between neuromuscular electrical stimulation and upper-extremity function in patients with stroke. *Stroke* 2010; 41: 821-824. 2010/03/06. DOI: 10.1161/strokeaha.109.574160.

83. Chae J, Bethoux F, Bohine T, et al. Neuromuscular stimulation for upper extremity motor and functional recovery in acute hemiplegia. *Stroke* 1998; 29: 975-979. 1998/05/22. DOI: 10.1161/01.str.29.5.975.

84. Tian R, Zhang B and Zhu Y. Rhythmic Auditory Stimulation as an Adjuvant Therapy Improved Post-stroke Motor Functions of the Upper Extremity: A Randomized Controlled Pilot Study. *Front Neurosci* 2020; 14: 649. 2020/07/28. DOI: 10.3389/fnins.2020.00649.

85. Chouhan S and Kumar S. Comparing the effects of rhythmic auditory cueing and visual cueing in acute hemiparetic stroke. *Int J Ther Rehabil* 2012; 19: 344-351.

86. Lee H-C, Kuo F-L, Lin Y-N, et al. Effects of robot-assisted rehabilitation on hand function of people with stroke: A randomized, crossover-controlled, assessor-blinded study. *Am J Occup Ther* 2021; 75: 7501205020p7501205021-7501205020p7501205011.

87. Jiang S, You H, Zhao W, et al. Effects of short-term upper limb robot-assisted therapy on the rehabilitation of sub-acute stroke patients. *Technol Health Care* 2021; 29: 295-303. 2020/12/09. DOI: 10.3233/thc-202127.

88. Ranzani R, Lambercy O, Metzger J-C, et al. Neurocognitive robot-assisted rehabilitation of hand function: a randomized control trial on motor recovery in subacute stroke. *J Neuroeng Rehabil* 2020; 17: 1-13.

89. Xu Q, Li C, Pan Y, et al. Impact of smart force feedback rehabilitation robot training on upper limb motor function in the subacute stage of stroke. *NeuroRehabilitation* 2020; 47: 209-215. 2020/08/04. DOI: 10.3233/nre-203130.

90. Calabro RS, Accorinti M, Porcari B, et al. Does hand robotic rehabilitation improve motor function by rebalancing interhemispheric connectivity after chronic stroke? Encouraging data from a randomised-clinical-trial. *Clin Neurophysiol* 2019; 130: 767-780.

91. Dehem S, Gilliaux M, Stoquart G, et al. Effectiveness of upper-limb robotic-assisted therapy in the early rehabilitation phase after stroke: A single-blind, randomised, controlled trial. *Ann Phys Rehabil Med* 2019; 62: 313-320.

92. Rodgers H, Bosomworth H, Krebs HI, et al. Robot assisted training for the upper limb after stroke (RATULS): a multicentre randomised controlled trial. *Lancet* 2019; 394: 51-62.

93. Lee M-J, Lee J-H and Lee S-M. Effects of robot-assisted therapy on upper extremity function and activities of daily living in hemiplegic patients: A single-blinded, randomized, controlled trial. *Technol Health Care* 2018; 26: 659-666.

94. Daunoraviciene K, Adomaviciene A, Grigonyte A, et al. Effects of robot-assisted training on upper limb functional recovery during the rehabilitation of poststroke patients. *Technol Health Care* 2018; 26: 533-542. 2018/05/31. DOI: 10.3233/thc-182500.

95. Tomić TJ, Savić AM, Vidaković AS, et al. ArmAssist Robotic System versus Matched Conventional Therapy for Poststroke Upper Limb Rehabilitation: A Randomized Clinical Trial. *Biomed Res Int* 2017; 2017: 7659893. 2017/03/03. DOI: 10.1155/2017/7659893.

96. Fan Y-t, Lin K-c, Liu H-l, et al. Neural correlates of motor recovery after robot-assisted stroke rehabilitation: a case series study. *Neurocase* 2016; 22: 416-425.

97. Susanto EA, Tong RK, Ockenfeld C, et al. Efficacy of robot-assisted fingers training in chronic stroke survivors: a pilot randomized-controlled trial. *J Neuroeng Rehabil* 2015; 12: 1-9.

98. Prange GB, Kottink AI, Buurke JH, et al. The effect of arm support combined with rehabilitation games on upper-extremity function in subacute stroke: a randomized controlled trial. *Neurorehabil Neural Repair* 2015; 29: 174-182. 2014/06/01. DOI: 10.1177/1545968314535985.

99. Masiero S, Armani M, Ferlini G, et al. Randomized trial of a robotic assistive device for the upper extremity during early inpatient stroke rehabilitation. *Neurorehabil Neural Repair* 2014; 28: 377-386. 2013/12/10. DOI: 10.1177/1545968313513073.

100. Klamroth-Marganska V, Blanco J, Campen K, et al. Three-dimensional, task-specific robot therapy of the arm after stroke: a multicentre, parallel-group randomised trial. *Lancet Neurol* 2014; 13: 159-166.

101. Ang KK, Guan C, Phua KS, et al. Brain-computer interface-based robotic end effector system for wrist and hand rehabilitation: results of a three-armed randomized controlled trial for chronic stroke. *Front Neuroeng* 2014; 7: 30.

102. Brokaw EB, Nichols D, Holley RJ, et al. Robotic therapy provides a stimulus for upper limb motor recovery after stroke that is complementary to and distinct from conventional therapy. *Neurorehabil Neural Repair* 2014; 28: 367-376.

103. Bartolo M, De Nunzio AM, Sebastiano F, et al. Arm weight support training improves functional motor outcome and movement smoothness after stroke. *Funct Neurol* 2014; 29: 15.

104. Yang C-L, Lin K-C, Chen H-C, et al. Pilot comparative study of unilateral and bilateral robot-assisted training on upper-extremity performance in patients with stroke. *Am J Occup Ther* 2012; 66: 198-206.

105. Reinkensmeyer DJ, Wolbrecht ET, Chan V, et al. Comparison of three-dimensional, assist-as-needed robotic arm/hand movement training provided with Pneu-WREX to conventional tabletop therapy after chronic stroke. *Am J Phys Med Rehabil* 2012; 91: S232-S241.

106. Conroy SS, Whitall J, Dipietro L, et al. Effect of gravity on robot-assisted motor training after chronic stroke: a randomized trial. *Arch Phys Med Rehabil* 2011; 92: 1754-1761.

107. Carmeli E, Peleg S, Bartur G, et al. HandTutorTM enhanced hand rehabilitation after stroke—a pilot study. *Physiother Res Int* 2011; 16: 191-200.

108. Masiero S and Armani M. Upper-limb robot-assisted therapy in rehabilitation of acute stroke patients: focused review and results of new randomized controlled trial. *J Rehabil Res Dev* 2011; 48: 355.

109. Lo AC, Guarino PD, Richards LG, et al. Robot-assisted therapy for long-term upper-limb impairment after stroke. *N Engl J Med* 2010; 362: 1772-1783.

110. Housman SJ, Scott KM and Reinkensmeyer DJ. A randomized controlled trial of gravity-supported, computer-enhanced arm exercise for individuals with severe hemiparesis. *Neurorehabil Neural Repair* 2009; 23: 505-514.

111. Lum PS, Burgar CG, Van der Loos M, et al. The MIME robotic system for upper-limb neuro-rehabilitation: results from a clinical trial in subacute stroke. In: *9th ICORR 2005*  2005, pp.511-514. IEEE.

112. Lum PS, Burgar CG, Shor PC, et al. Robot-assisted movement training compared with conventional therapy techniques for the rehabilitation of upper-limb motor function after stroke. *Arch Phys Med Rehabil* 2002; 83: 952-959.

113. Derakhshanfar M, Raji P, Bagheri H, et al. Sensory interventions on motor function, activities of daily living, and spasticity of the upper limb in people with stroke: A randomized clinical trial. *J Hand Ther* 2021; 34: 515-520. 2020/06/23. DOI: 10.1016/j.jht.2020.03.028.

114. de Diego C, Puig S and Navarro X. A sensorimotor stimulation program for rehabilitation of chronic stroke patients. *Restor Neurol Neurosci* 2013; 31: 361-371. 2013/03/26. DOI: 10.3233/rnn-120250.

115. Arya KN, Verma R, Garg RK, et al. Meaningful task-specific training (MTST) for stroke rehabilitation: a randomized controlled trial. *Top Stroke Rehabil* 2012; 19: 193-211. 2012/06/07. DOI: 10.1310/tsr1903-193.

116. Winstein CJ, Rose DK, Tan SM, et al. A randomized controlled comparison of upper-extremity rehabilitation strategies in acute stroke: A pilot study of immediate and long-term outcomes. *Arch Phys Med Rehabil* 2004; 85: 620-628. 2004/04/15. DOI: 10.1016/j.apmr.2003.06.027.

117. Nelles G, Jentzen W, Jueptner M, et al. Arm training induced brain plasticity in stroke studied with serial positron emission tomography. *Neuroimage* 2001; 13: 1146-1154. 2001/05/16. DOI: 10.1006/nimg.2001.0757.

118. Khan F, Rathore C, Kate M, et al. The comparative efficacy of theta burst stimulation or functional electrical stimulation when combined with physical therapy after stroke: a randomized controlled trial. *Clin Rehabil* 2019; 33: 693-703. 2019/01/09. DOI: 10.1177/0269215518820896.

119. Hsu YF, Huang YZ, Lin YY, et al. Intermittent theta burst stimulation over ipsilesional primary motor cortex of subacute ischemic stroke patients: a pilot study. *Brain Stimul* 2013; 6: 166-174. 2012/06/05. DOI: 10.1016/j.brs.2012.04.007.

120. Wu D, Ma J, Zhang L, et al. Effect and Safety of Transcutaneous Auricular Vagus Nerve Stimulation on Recovery of Upper Limb Motor Function in Subacute Ischemic Stroke Patients: A Randomized Pilot Study. *Neural Plast* 2020; 2020: 8841752. 2020/08/18. DOI: 10.1155/2020/8841752.

121. Mekbib DB, Debeli DK, Zhang L, et al. A novel fully immersive virtual reality environment for upper extremity rehabilitation in patients with stroke. *Ann N Y Acad Sci* 2021; 1493: 75-89. 2021/01/15. DOI: 10.1111/nyas.14554.

122. Marques-Sule E, Arnal-Gómez A, Buitrago-Jiménez G, et al. Effectiveness of Nintendo Wii and Physical Therapy in Functionality, Balance, and Daily Activities in Chronic Stroke Patients. *J Am Med Dir Assoc* 2021; 22: 1073-1080. 2021/02/28. DOI: 10.1016/j.jamda.2021.01.076.

123. Laffont I, Froger J, Jourdan C, et al. Rehabilitation of the upper arm early after stroke: Video games versus conventional rehabilitation. A randomized controlled trial. *Ann Phys Rehabil Med* 2020; 63: 173-180. 2019/12/13. DOI: 10.1016/j.rehab.2019.10.009.

124. Kang MG, Yun SJ, Lee SY, et al. Effects of Upper-Extremity Rehabilitation Using Smart Glove in Patients With Subacute Stroke: Results of a Prematurely Terminated Multicenter Randomized Controlled Trial. *Front Neurol* 2020; 11: 580393. 2020/11/27. DOI: 10.3389/fneur.2020.580393.

125. Long Y, Ouyang RG and Zhang JQ. Effects of virtual reality training on occupational performance and self-efficacy of patients with stroke: a randomized controlled trial. *J Neuroeng Rehabil* 2020; 17: 150. 2020/11/15. DOI: 10.1186/s12984-020-00783-2.

126. Norouzi-Gheidari N, Hernandez A, Archambault PS, et al. Feasibility, Safety and Efficacy of a Virtual Reality Exergame System to Supplement Upper Extremity Rehabilitation Post-Stroke: A Pilot Randomized Clinical Trial and Proof of Principle. *Int J Environ Res Public Health* 2020; 17: 113. 2019/12/28. DOI: 10.3390/ijerph17010113.

127. Keskin Y, Atci AG, Urkmez B, et al. Efficacy of a video-based physical therapy and rehabilitation system in patients with post-stroke hemiplegia: A randomized controlled pilot trial. *Türk Geriatri Dergisi* 2020; 23.

128. Park M, Ko MH, Oh SW, et al. Effects of virtual reality-based planar motion exercises on upper extremity function, range of motion, and health-related quality of life: a multicenter, single-blinded, randomized, controlled pilot study. *J Neuroeng Rehabil* 2019; 16: 122. 2019/10/28. DOI: 10.1186/s12984-019-0595-8.

129. Oh YB, Kim GW, Han KS, et al. Efficacy of Virtual Reality Combined With Real Instrument Training for Patients With Stroke: A Randomized Controlled Trial. *Arch Phys Med Rehabil* 2019; 100: 1400-1408. 2019/04/20. DOI: 10.1016/j.apmr.2019.03.013.

130. Ögün MN, Kurul R, Yaşar MF, et al. Effect of Leap Motion-based 3D Immersive Virtual Reality Usage on Upper Extremity Function in Ischemic Stroke Patients. *Arq Neuropsiquiatr* 2019; 77: 681-688. 2019/10/31. DOI: 10.1590/0004-282x20190129.

131. Henrique PPB, Colussi EL and De Marchi ACB. Effects of Exergame on Patients' Balance and Upper Limb Motor Function after Stroke: A Randomized Controlled Trial. *J Stroke Cerebrovasc Dis* 2019; 28: 2351-2357. 2019/06/18. DOI: 10.1016/j.jstrokecerebrovasdis.2019.05.031.

132. Hung JW, Chou CX, Chang YJ, et al. Comparison of Kinect2Scratch game-based training and therapist-based training for the improvement of upper extremity functions of patients with chronic stroke: a randomized controlled single-blinded trial. *Eur J Phys Rehabil Med* 2019; 55: 542-550. 2019/02/21. DOI: 10.23736/s1973-9087.19.05598-9.

133. Ikbali Afsar S, Mirzayev I, Umit Yemisci O, et al. Virtual Reality in Upper Extremity Rehabilitation of Stroke Patients: A Randomized Controlled Trial. *J Stroke Cerebrovasc Dis* 2018; 27: 3473-3478. 2018/09/09. DOI: 10.1016/j.jstrokecerebrovasdis.2018.08.007.

134. Faria AL, Cameirão MS, Couras JF, et al. Combined Cognitive-Motor Rehabilitation in Virtual Reality Improves Motor Outcomes in Chronic Stroke - A Pilot Study. *Front Psychol* 2018; 9: 854. 2018/06/15. DOI: 10.3389/fpsyg.2018.00854.

135. Kim WS, Cho S, Park SH, et al. A low cost kinect-based virtual rehabilitation system for inpatient rehabilitation of the upper limb in patients with subacute stroke: A randomized, double-blind, sham-controlled pilot trial. *Medicine (Baltimore)* 2018; 97: e11173. 2018/06/21. DOI: 10.1097/md.0000000000011173.

136. Kiper P, Szczudlik A, Agostini M, et al. Virtual Reality for Upper Limb Rehabilitation in Subacute and Chronic Stroke: A Randomized Controlled Trial. *Arch Phys Med Rehabil* 2018; 99: 834-842.e834. 2018/02/18. DOI: 10.1016/j.apmr.2018.01.023.

137. Aşkın A, Atar E, Koçyiğit H, et al. Effects of Kinect-based virtual reality game training on upper extremity motor recovery in chronic stroke. *Somatosens Mot Res* 2018; 35: 25-32. 2018/03/14. DOI: 10.1080/08990220.2018.1444599.

138. Kong KH, Loh YJ, Thia E, et al. Efficacy of a Virtual Reality Commercial Gaming Device in Upper Limb Recovery after Stroke: A Randomized, Controlled Study. *Top Stroke Rehabil* 2016; 23: 333-340. 2016/04/22. DOI: 10.1080/10749357.2016.1139796.

139. Lee MM, Shin DC and Song CH. Canoe game-based virtual reality training to improve trunk postural stability, balance, and upper limb motor function in subacute stroke patients: a randomized controlled pilot study. *J Phys Ther Sci* 2016; 28: 2019-2024. 2016/08/12. DOI: 10.1589/jpts.28.2019.

140. Seok H, Lee SY, Kim J, et al. Can Short-Term Constraint-Induced Movement Therapy Combined With Visual Biofeedback Training Improve Hemiplegic Upper Limb Function of Subacute Stroke Patients? *Ann Rehabil Med* 2016; 40: 998-1009. 2017/01/26. DOI: 10.5535/arm.2016.40.6.998.

141. Shin JH, Kim MY, Lee JY, et al. Effects of virtual reality-based rehabilitation on distal upper extremity function and health-related quality of life: a single-blinded, randomized controlled trial. *J Neuroeng Rehabil* 2016; 13: 17. 2016/02/26. DOI: 10.1186/s12984-016-0125-x.

142. da Silva Ribeiro NM, Ferraz DD, Pedreira É, et al. Virtual rehabilitation via Nintendo Wii® and conventional physical therapy effectively treat post-stroke hemiparetic patients. *Top Stroke Rehabil* 2015; 22: 299-305. 2015/08/11. DOI: 10.1179/1074935714z.0000000017.

143. Shin JH, Bog Park S and Ho Jang S. Effects of game-based virtual reality on health-related quality of life in chronic stroke patients: A randomized, controlled study. *Comput Biol Med* 2015; 63: 92-98. 2015/06/06. DOI: 10.1016/j.compbiomed.2015.03.011.

144. Shin JH, Ryu H and Jang SH. A task-specific interactive game-based virtual reality rehabilitation system for patients with stroke: a usability test and two clinical experiments. *J Neuroeng Rehabil* 2014; 11: 32. 2014/03/07. DOI: 10.1186/1743-0003-11-32.

145. Choi JH, Han EY, Kim BR, et al. Effectiveness of commercial gaming-based virtual reality movement therapy on functional recovery of upper extremity in subacute stroke patients. *Ann Rehabil Med* 2014; 38: 485-493. 2014/09/18. DOI: 10.5535/arm.2014.38.4.485.

146. Yin CW, Sien NY, Ying LA, et al. Virtual reality for upper extremity rehabilitation in early stroke: a pilot randomized controlled trial. *Clin Rehabil* 2014; 28: 1107-1114. 2014/05/08. DOI: 10.1177/0269215514532851.

147. Kottink AI, Prange GB, Krabben T, et al. Gaming and Conventional Exercises for Improvement of Arm Function After Stroke: A Randomized Controlled Pilot Study. *Games Health J* 2014; 3: 184-191. 2015/07/22. DOI: 10.1089/g4h.2014.0026.

148. Kiper P, Agostini M, Luque-Moreno C, et al. Reinforced feedback in virtual environment for rehabilitation of upper extremity dysfunction after stroke: preliminary data from a randomized controlled trial. *Biomed Res Int* 2014; 2014: 752128. 2014/04/20. DOI: 10.1155/2014/752128.

149. Friedman N, Chan V, Reinkensmeyer AN, et al. Retraining and assessing hand movement after stroke using the MusicGlove: comparison with conventional hand therapy and isometric grip training. *J Neuroeng Rehabil* 2014; 11: 76. 2014/06/03. DOI: 10.1186/1743-0003-11-76.

150. Sin H and Lee G. Additional virtual reality training using Xbox Kinect in stroke survivors with hemiplegia. *Am J Phys Med Rehabil* 2013; 92: 871-880. 2013/09/21. DOI: 10.1097/PHM.0b013e3182a38e40.

151. Duff M, Chen Y, Cheng L, et al. Adaptive mixed reality rehabilitation improves quality of reaching movements more than traditional reaching therapy following stroke. *Neurorehabil Neural Repair* 2013; 27: 306-315. 2012/12/06. DOI: 10.1177/1545968312465195.

152. Kwon JS, Park MJ, Yoon IJ, et al. Effects of virtual reality on upper extremity function and activities of daily living performance in acute stroke: a double-blind randomized clinical trial. *NeuroRehabilitation* 2012; 31: 379-385. 2012/12/13. DOI: 10.3233/nre-2012-00807.

153. Levin MF, Snir O, Liebermann DG, et al. Virtual reality versus conventional treatment of reaching ability in chronic stroke: clinical feasibility study. *Neurol Ther* 2012; 1: 3. 2012/12/01. DOI: 10.1007/s40120-012-0003-9.

154. In TS, Jung KS, Lee SW, et al. Virtual reality reflection therapy improves motor recovery and motor function in the upper extremities of people with chronic stroke. *J Phys Ther Sci* 2012; 24: 339-343.

155. Kiper P, Piron L, Turolla A, et al. The effectiveness of reinforced feedback in virtual environment in the first 12 months after stroke. *Neurol Neurochir Pol* 2011; 45: 436-444. 2011/12/01. DOI: 10.1016/s0028-3843(14)60311-x.

156. Piron L, Turolla A, Agostini M, et al. Motor learning principles for rehabilitation: a pilot randomized controlled study in poststroke patients. *Neurorehabil Neural Repair* 2010; 24: 501-508. 2010/06/29. DOI: 10.1177/1545968310362672.

157. Piron L, Turolla A, Agostini M, et al. Exercises for paretic upper limb after stroke: a combined virtual-reality and telemedicine approach. *J Rehabil Med* 2009; 41: 1016-1102. 2009/10/21. DOI: 10.2340/16501977-0459.
