# Supplementary material for: Time Post-Stroke and Upper Extremity Stroke Motor Recovery Rehabilitation: A Meta-Analysis
Source: Neurorehabil Neural Repair. 2025 Jul 23;39(11):945–53. doi: 10.1177/15459683251356975 (PMC12531395; doi:10.1177/15459683251356975)
Supplement: sj-pdf-2-nnr-10.1177_15459683251356975 – Supplemental material for Time Post-Stroke and Upper Extremity Stroke Motor Recovery Rehabilitation: A Meta-Analysis [file sj-pdf-2-nnr-10.1177_15459683251356975.pdf]

Supplemental File 2

**Risk of Bias Domains**
D1: Random Sequence Generation
D2: Allocation Concealment
D3: Blinding of Participants and Personnel
D4: Blinding of Outcome Assessment
D5: Incomplete Outcome Data
D6: Selective Reporting
D7: Other Source of Bias

(red)	**High**
(yellow)	**Unclear**
(green)	**Low**

**Study**	**Domain 1**	**Domain 2**	**Domain 3**	**Domain 4**	**Domain 5**	**Domain 6**	**Domain 7**	**Overall Risk**
Alexander 2004	Unclear	Unclear	High	Low	Low	Unclear	Low	High
Wang 2020	Low	Low	Low	Low	Low	Unclear	Low	Unclear
Hsieh 2007	Low	Low	High	Low	Low	Unclear	Low	High
Zhang 2017	Low	Low	High	Low	Low	Unclear	Low	High
Kim 2020	Low	Low	High	Low	Low	Low	Low	High
Fu 2017	Low	Unclear	Unclear	High	Low	Unclear	Low	High
Zhu 2015	Low	Unclear	Unclear	Low	Low	Unclear	Low	Unclear
Zhu 2020	Unclear	Unclear	Low	Low	Low	Unclear	Low	Unclear
Desrosiers 2005	Low	Low	Low	Low	Low	Unclear	Low	Unclear
Lee 2017	Low	Unclear	Unclear	Low	Low	Unclear	Low	Unclear
Lin 2010a	Unclear	Unclear	Unclear	Low	Low	Unclear	Low	Unclear
Lee 2019	Unclear	Unclear	Unclear	Unclear	Low	Unclear	Low	Unclear

Lin 2009a								
Lin 2015								
Lee & Lee 2015								
Alisar 2020								
Nair 2011								
Fusco 2014								
Wu 2013a								
Abdullahi 2018								
Yoon 2014								
Thrane 2015								
Boake 2007								
El-He3 2015								
Singh 2013								
Page 2005								
vanDelden 2013								
Yadav 2016								
Wu 2007a								
Lin 2008								
Lin 2009b								
Lin 2010b								
Page 2008								
Wu 2007b								
Wu 2012								
Yang 2017								
Tosun 2017								
El-Tamawy 2019								
Seniow 2012								
Sharma 2020								
Li 2016								
Long 2018								
Hosomi 2016								

BarrosGalvao 2014								
Harvey 2018								
Etoh 2013								
Askin 2017								
Ibrahim 2020								
Guan 2017								
Wu 2013b								
Kim 2016								
Chinnavan 2020								
Colomer 2016								
Arya 2015								
Michielsen 2011								
Ding 2018								
Guo 2019								
MirelaCristina 2015								
Samuelkamaleshkumar 2014								
Lee 2012								
Bai 2019								
Thieme 2013								
Antoniotti 2019								
Lim 2016								
Gurbuz 2016								
Chan 2018								
Madhoun 2020								
Ding 2019								
Page 2001								
Sun 2013								
Wang 2019								
Kim 2015								
Oh 2016								
Nam 2019								

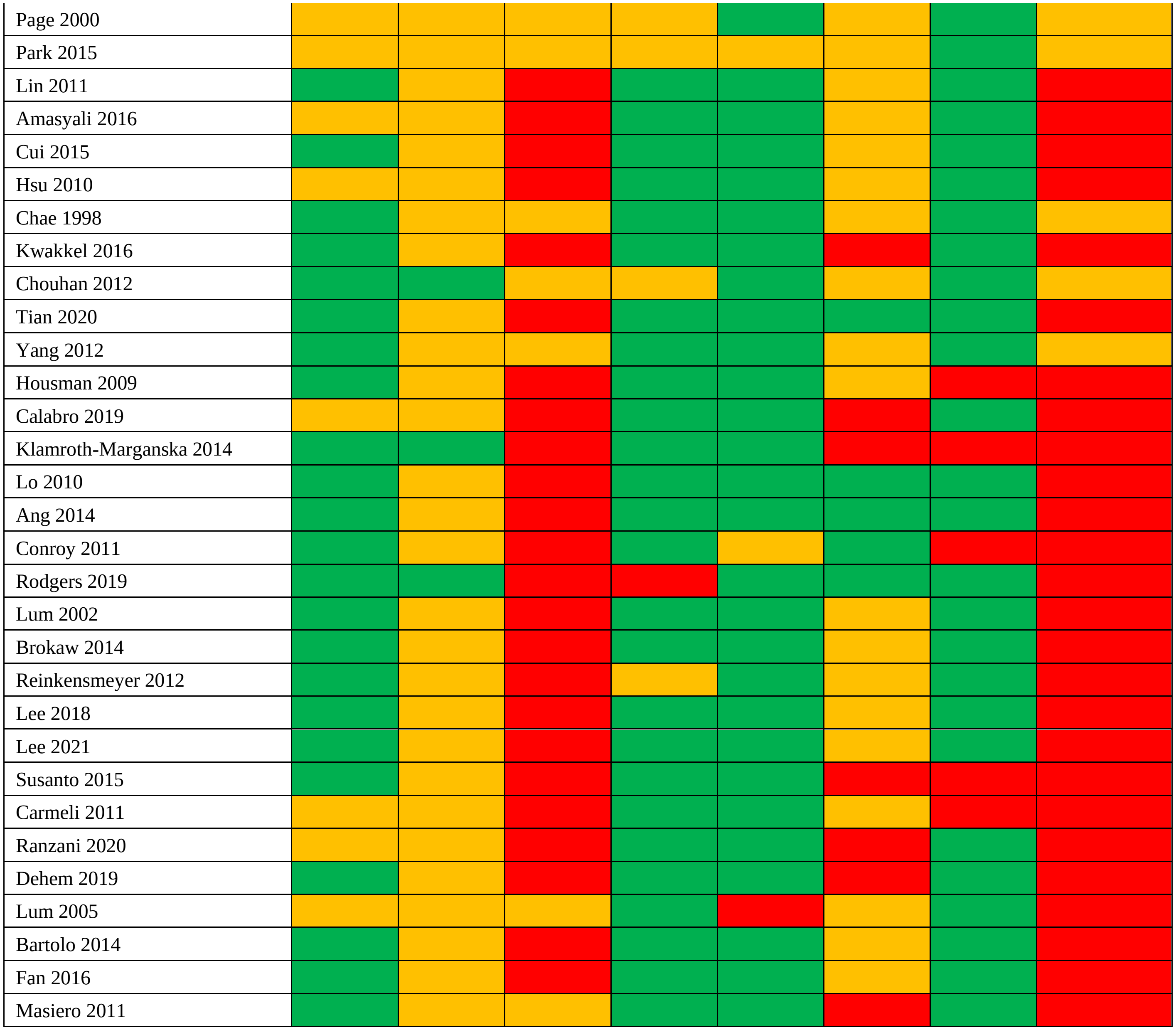
Page 2000
Park 2015
Lin 2011
Amasyali 2016
Cui 2015
Hsu 2010
Chae 1998
Kwakkel 2016
Chouhan 2012
Tian 2020
Yang 2012
Housman 2009
Calabro 2019
Klamroth-Marganska 2014
Lo 2010
Ang 2014
Conroy 2011
Rodgers 2019
Lum 2002
Brokaw 2014
Reinkensmeyer 2012
Lee 2018
Lee 2021
Susanto 2015
Carmeli 2011
Ranzani 2020
Dehem 2019
Lum 2005
Bartolo 2014
Fan 2016
Masiero 2011

Xu 2020								
Jiang 2021								
Tomic 2017								
Daunoraviciene 2018								
Prange 2015								
Masiero 2014								
deDiego 2013								
Derakhshanfar 2020								
Nelles 2001								
Arya 2012								
Winstein 2004								
Hsu 2013								
Khan 2019								
Zhou 2018								
Wu 2020								
Laffont 2020								
Kong 2016								
Lee 2016								
IkbaliAfsar 2018								
Shin 2014								
Choi 2014								
Kwon 2012								
Mekbib 2021								
Seok 2016								
Kim 2018								
Kiper 2011								
Kiper 2018								
Kang 2020								
Yin 2014								
Long 2020								
daSilvaRibeiro 2015								

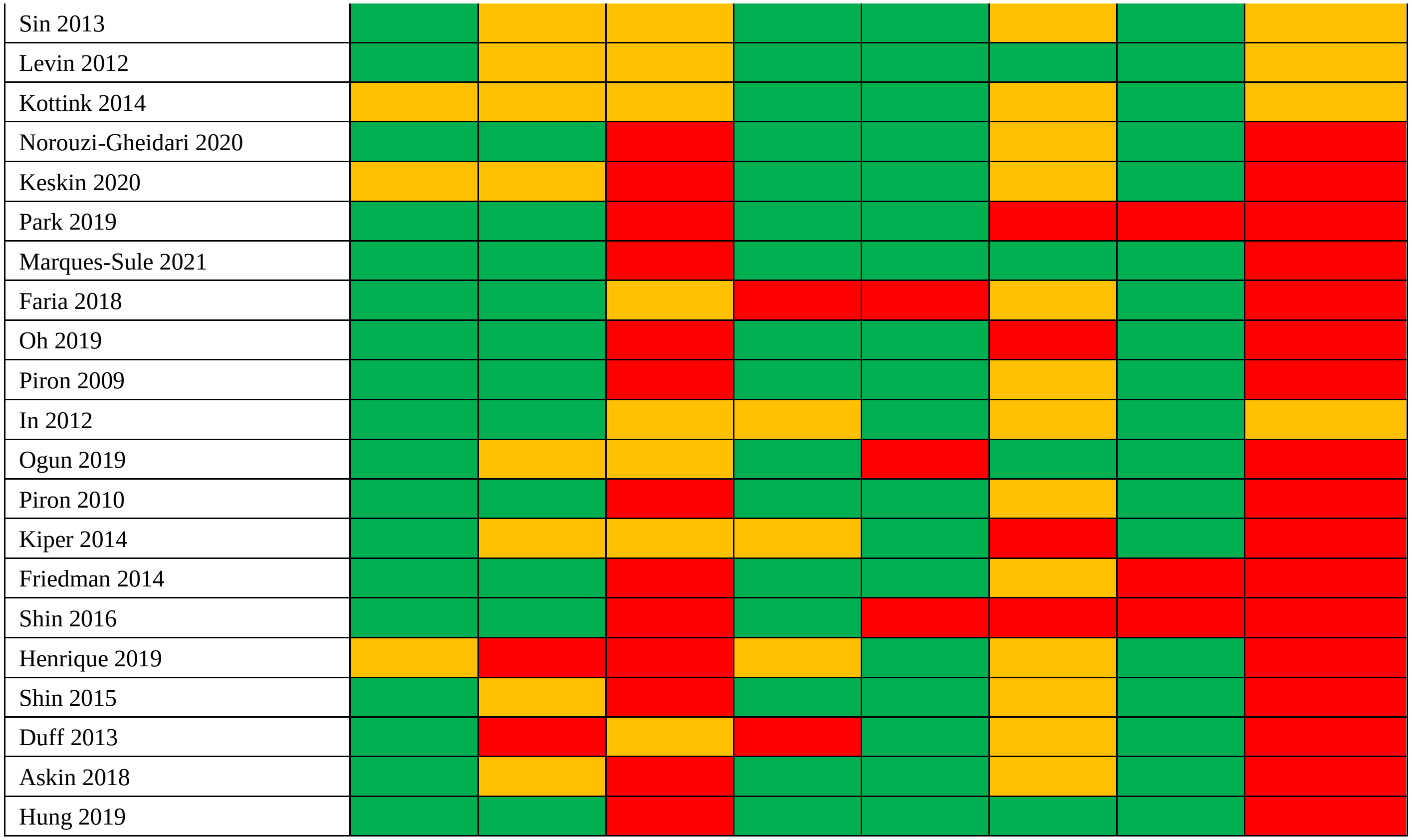

**RoB**	**Low**	**Unclear**	**High**
D1	76.4%	23.6%	0.0%
D2	36.3%	61.8%	1.9%
D3	10.8%	35.1%	54.1%
D4	77.7%	17.2%	5.1%
D5	92.4%	4.5%	3.2%
D6	20.4%	67.5%	12.1%
D7	92.4%	0.0%	7.6%

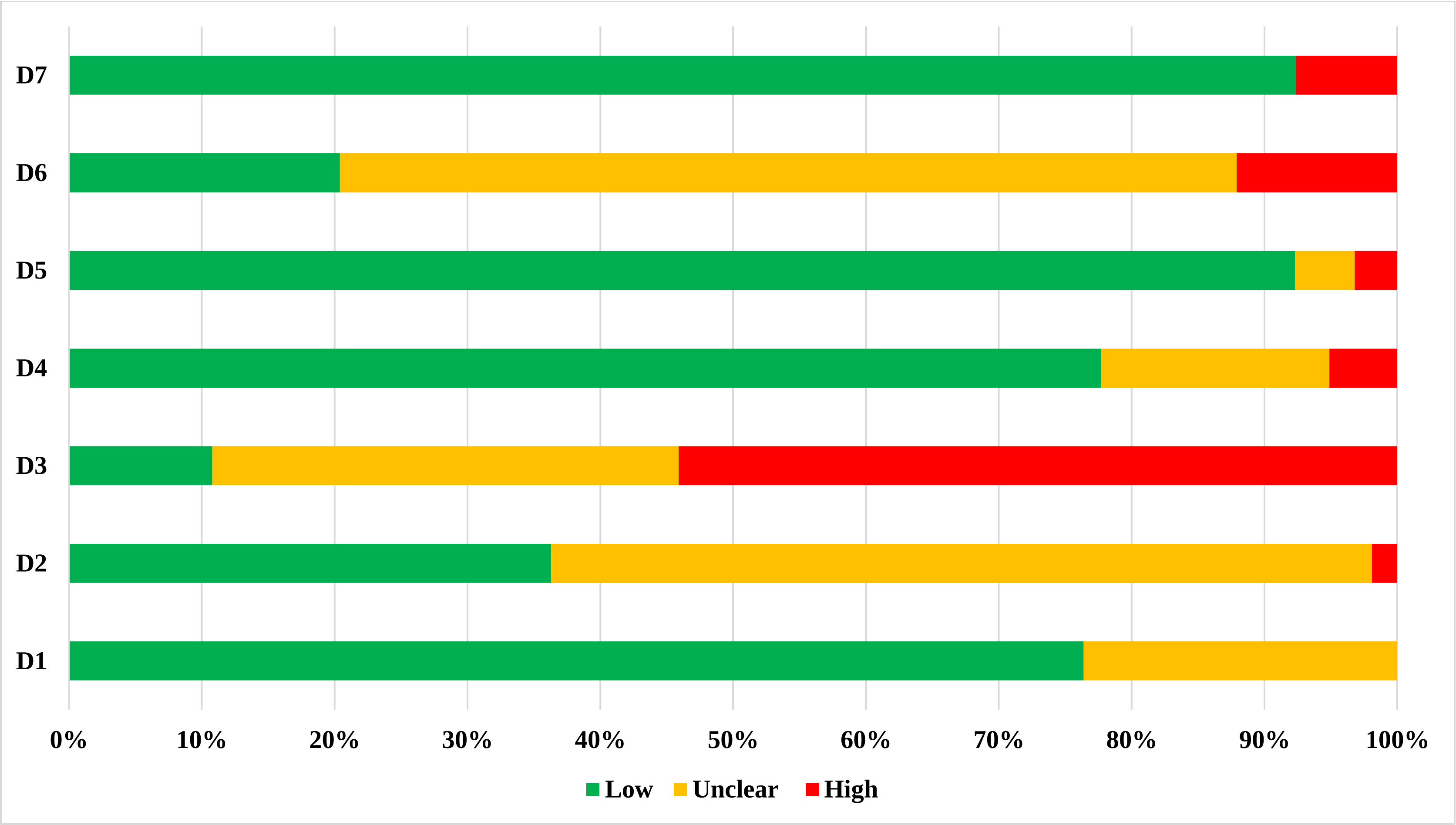
D7
D6
D5
D4
D3
D2
D1
0%
10%
20%
30%
40%
50%
60%
70%
80%
90%
100%
Low
Unclear
High
